# Combinatorial Antibacterial and Antibiofilm Activities of Medicinal Plant Extracts Against Oral Bacterial Pathogens

**DOI:** 10.3390/microorganisms14102228

**Published:** 2026-10-02

**Authors:** Mulanga Luscious Mulaudzi, Ashwell Rungano Ndhlala, Peter Masoko

**Affiliations:** 1 Department of Biochemistry, Microbiology and Biotechnology, University of Limpopo, Private Bag X1106, Sovenga 0727, South Africa; luscious.mulaudzi@ul.ac.za; 2 Department of Plant Production, Soil Science and Agricultural Engineering, Green Biotechnologies Research Centre of Excellence, University of Limpopo, Private Bag X1106, Sovenga 0727, South Africa; ashwell.ndhlala@ul.ac.za

**Keywords:** antibacterial, biofilm, oral health, plant extract, phytochemicals

## Abstract

This study investigated the antibacterial properties and biofilm disruption mechanisms of five medicinal plants traditionally used in Limpopo communities for the treatment of oral infections, namely *Sesamum senecioides* Klotzsch, *Euclea undulata* Thunb, *Kleinia longiflora* DC, *Aloe greatheadii* Schönland, and *Drimia elata* Jacq. Five plants were analysed using four solvents (methanol, dichloromethane (DCM), petroleum ether (PE), and water) of different polarities to quantify their phytochemical constituents. Antibacterial, combinational effects, and antibiofilm activities were evaluated through broth microdilution and crystal violet staining assays. Phytochemical analysis revealed that *E. undulata* had the highest levels of tannins (2400.74 ± 8.31 mg GAE/g in petroleum ether), phenolic compounds (377.63 ± 2.25 mg GAE/g), and flavonoids (31.26 ± 2.76 mg QE/g). It also demonstrated low minimum inhibitory concentrations (MICs) of 0.15 µg/mL against *Streptococcus mutans* and *Porphyromonas gingivalis.* Combination studies showed that *E. undulata* and *K. longiflora* exhibited a synergistic antibacterial effect (FICI = 0.15) at the tested concentrations. In the biofilm assays, *E. undulata* inhibited more than 60% of biofilm formation, while *A. greatheadii* exhibited strong antibiofilm activity, with inhibition exceeding 60% across several concentrations tested. During the initial biofilm prevention stage, inhibition was exhibited against *S. mutans* at 1.26 and 2.50 µg/mL and against *P. gingivalis* at 1.24 µg/mL. At the initial cell attachment stage, *A. greatheadii* exhibited over 60% inhibition of *P. gingivalis* at 1.24 µg/mL. During the biofilm eradication stage, inhibition exceeding 60% was observed against *Pseudomonas aeruginosa* at 1.24 µg/mL, *S. mutans* across 0.63–2.52 µg/mL and *P. gingivalis* across 0.62–1.24 µg/mL. Overall, these findings demonstrate the antibacterial and antibiofilm potential of the investigated medicinal plants against dental pathogens and support their further investigation as potential sources of bioactive compounds for alternative approaches to managing dental infections.

## 1. Introduction

Oral infections are among the most prevalent chronic diseases worldwide and represent a major public health burden due to their association with pain, tooth loss, reduced quality of life, and systemic complications [1]. These infections are largely mediated by polymicrobial biofilms, which are structured microbial communities enclosed in an extracellular polymeric matrix that enhances microbial adhesion, persistence, and resistance to antibacterial agents [2]. Dental plaque is one of the most clinically significant oral biofilms and is strongly associated with dental caries, periodontitis, and endodontic infections. *Streptococcus mutans* is regarded as a major cariogenic pathogen because of its ability to adhere to tooth surfaces, synthesise extracellular polysaccharides, and produce acids that demineralise dental enamel. In contrast, *Porphyromonas gingivalis* functions as a keystone periodontal pathogen capable of dysregulating host immune responses and promoting chronic inflammatory destruction of periodontal tissues [3].

In addition to these classical oral pathogens, opportunistic microorganisms such as *Enterococcus faecalis*, *Staphylococcus aureus*, and *Pseudomonas aeruginosa* have increasingly been associated with persistent endodontic infections, failed root canal treatments, and oral infections in immunocompromised individuals [4,5,6]. *E. faecalis* is particularly linked to refractory apical periodontitis due to its ability to survive nutrient deprivation, tolerate alkaline conditions, and form resistant biofilms within root canal systems [4]. Similarly, *S. aureus* and *P. aeruginosa* contribute to persistent inflammation and treatment failure in chronic oral lesions and secondary endodontic infections [5]. Biofilm-associated infections are therefore difficult to eradicate because microorganisms embedded within biofilms exhibit enhanced tolerance to antibacterial agents and host immune defences [2].

The growing emergence of antimicrobial resistance (AMR) among oral and opportunistic pathogens has further reduced the effectiveness of conventional antibiotics, leading to recurrent and difficult-to-treat infections [7,8]. Excessive and inappropriate use of antibacterials has accelerated the development of multidrug-resistant microorganisms, highlighting the urgent need for alternative therapeutic strategies capable of controlling both microbial growth and biofilm formation [9,10]. Medicinal plants have gained increasing attention as potential alternatives to conventional antibacterial therapies because they are rich sources of bioactive phytochemicals with antibacterial, antibiofilm, antioxidant, and anti-inflammatory properties [11,12]. Secondary metabolites such as flavonoids, tannins, alkaloids, terpenoids, and essential oils have demonstrated the ability to disrupt microbial membranes, inhibit quorum sensing, suppress virulence factors, and interfere with biofilm development [13,14]. Furthermore, recent studies suggest that combining phytochemicals with conventional antibiotics or with other plant-derived compounds may produce synergistic effects that enhance antibacterial efficacy while reducing the likelihood of resistance development [14]. Such synergistic interactions may lower the required doses of antibiotics, thereby minimising toxicity and adverse side effects.

Several traditional medicinal plants from southern Africa have shown potential for managing infections and inflammation. *Euclea undulata* is commonly used for toothache, headaches, fever, diabetes, and body pains [15,16]. Phytochemical investigations have identified alkaloids, flavonoids, phenolics, tannins, naphthoquinones, terpenes and phytosterols in *E. undulata*, while extracts have demonstrated antimicrobial, anti-inflammatory, antioxidant and hypoglycaemic activities [15]. *Kleinia longiflora* addresses reproductive conditions and inflamed gums [16]. The genus *Kleinia* contains diverse secondary metabolites, including flavonoids, alkaloids, saponins, terpenes and terpenoids, with reported antimicrobial, anti-inflammatory and other pharmacological activities [16]. *Aloe greatheadii* is applied for burns and skin issues, with insufficient documentation on its oral healthcare uses [17]. Studies of *A. greatheadii* have identified polyphenols, phenolic acids, phytosterols, fatty acids and other metabolites, and have demonstrated antioxidant activity; moreover, phytochemical composition varies according to geographical location, including populations from Limpopo Province [17,18,19]. *Drimia elata* is used for various ailments, including cardiovascular and respiratory issues [20]. Its medicinal relevance has been associated with its phenolic and flavonoid constituents and reported biological activities, although environmental factors may influence its phytochemical content and antibacterial efficacy. *Sesamum senecioides* treats abdominal pain, sexually transmitted infections (STIs), malaria, and reproductive health [21]. The plant contains saponins and has been traditionally associated with antibacterial and anti-inflammatory properties, supporting its potential for further investigation as a medicinal resource. Importantly, Bapedi traditional healers play a significant role in maintaining indigenous medicinal knowledge and providing healthcare within rural communities of Limpopo Province [22]. Ethnobotanical surveys have documented extensive use of medicinal plants by Bapedi healers for conditions including infections, reproductive disorders, diabetes and other health problems, highlighting the importance of this knowledge in guiding the scientific investigation of locally used plants [23]. The Ga-Molepo community, within Limpopo Province, similarly retains substantial indigenous knowledge of plant-based remedies, with recent research confirming continued use of indigenous plants for nutritional and medicinal purposes [24]. Despite their ethnomedicinal importance, many of these species are underexplored regarding phytochemical composition and antibiofilm potential against oral pathogens.

Therefore, this study hypothesised that selected medicinal plants traditionally used for healthcare applications possess significant antibacterial and antibiofilm activities against oral and opportunistic pathogens and that phytochemicals present in these plants may interact synergistically to enhance their antibacterial efficacy. To address this hypothesis, plant extracts were prepared using methanol, dichloromethane (DCM), petroleum ether, and water. The extracts were evaluated for their total phenolic, flavonoid, and tannin contents, while their phytochemical profiles were characterised using high-performance thin-layer chromatography (HPTLC). Antibacterial activity was assessed using the broth microdilution assay to determine the minimum inhibitory concentrations (MICs) against selected bacterial pathogens. The combinational effects of selected plant extracts were subsequently evaluated to determine potential synergistic, additive, or antagonistic interactions. In addition, antibiofilm activity was investigated by assessing the ability of the extracts to prevent biofilm formation, inhibit initial bacterial cell attachment, and eradicate preformed biofilms. Collectively, these approaches were used to establish relationships between the phytochemical composition of the extracts and their antibacterial and antibiofilm activities.

## 2. Materials and Methods

### 2.1. Plant Collection

**Figure 1:**
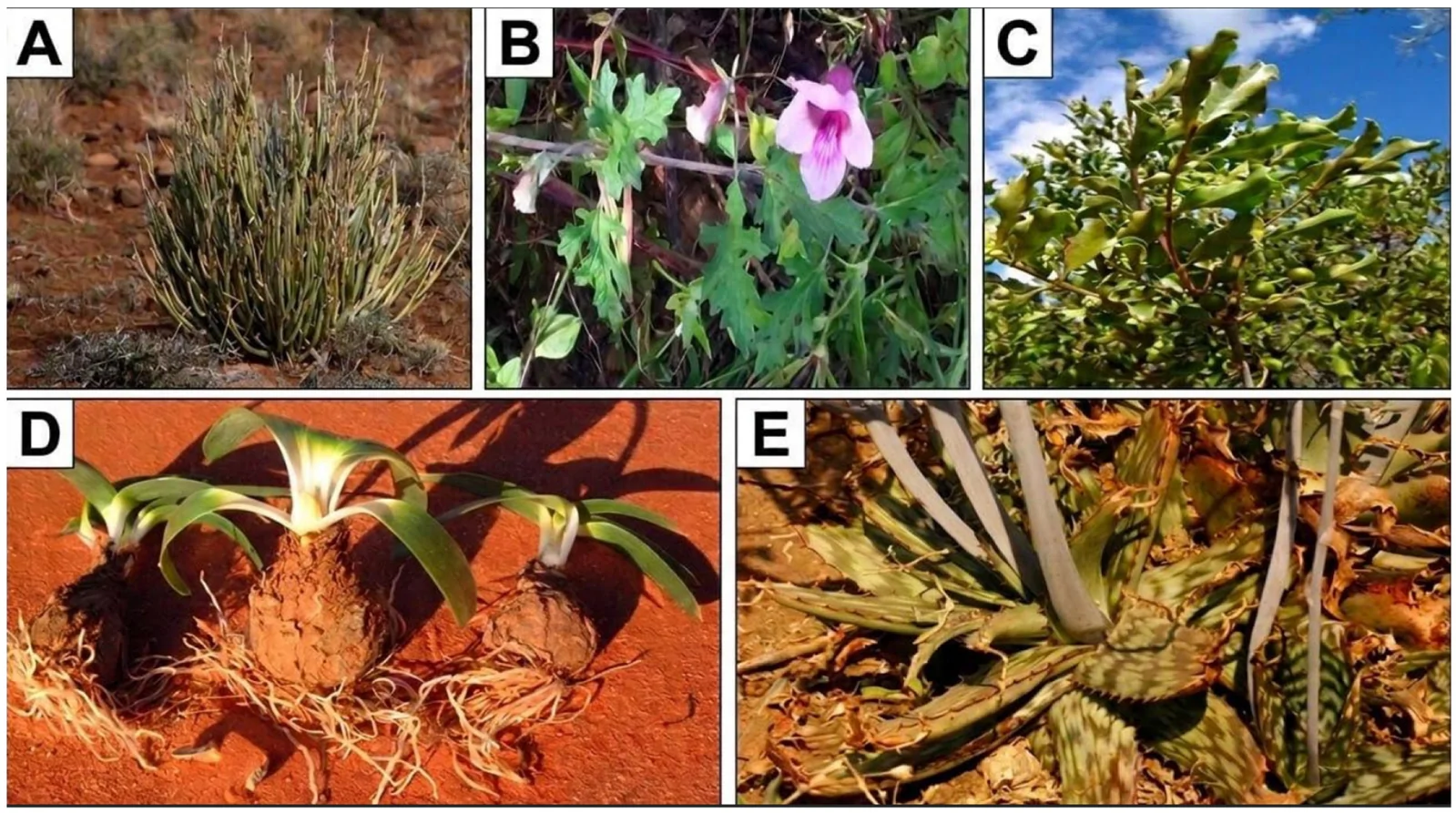
Selected medicinal plants investigated in the present study: (**A**) *Kleinia longiflora*, (**B**) *Sesamum senecioides*, (**C**) *Euclea undulata*, (**D**) *Drimia elata*, and (**E**) *Aloe greatheadii*. Images reproduced from SANBI PlantZAfrica and the Useful Tropical Plants Database, with acknowledgement of the South African National Biodiversity Institute (SANBI) and the respective photographers: (**A**) Dr Tony Rebelo, (**B**) Mashudu Nndanduleni, (**C**) Werner Voigt, (**D**) S. Rügheimer, and (**E**) Richard Morris; https://pza.sanbi.org/?https://tropical.theferns.info/?/ (accessed on 20 June 2026).

A systematic review process led to the selection of the five plants to be used in this current study [23]. All five selected medicinal plants were collected from the Ga-Molepo community and transported to the laboratory for further processing. The collected plant materials included the leaves of *Kleinia longiflora* (DC.) Haw., *Euclea undulata* Thunb., *Sesamum senecioides* (Klotzsch) Asch., and *Aloe greatheadii* var. *davyana* (Schönland) Glen & D.S.Hardy, as well as the bulbs of *Drimia elata* Jacq (Figure 1). Taxonomic identification of the plant species was conducted, and voucher specimens were deposited at the Herbarium (Table 1). The collected plant materials were shredded and shade-dried at ambient temperature before further processing. An electric grinder (Sundy hammer crusher SDHC 150, Sundy Science and Technology Co., Ltd., Changsha, China) was used to grind the dried plant materials to a powder, which was stored in closed cabinets until further use.

### 2.2. Extraction Preparation

The finely ground plant material (1 g), extracted with 10 mL of solvents with varying polarities, namely methanol (SupraSolv^®^, Darmstadt, Germany), dichloromethane (SupraSolv^®^, Darmstadt, Germany), petroleum ether (Merck, Johannesburg, South Africa, Cas no. 110-54-3), and water, was shaken at 200 rpm in a shaking incubator (Labotec model 20.2, LACOTEC GmbH, Reichshof, Germany) for 1 h. The solvents from the filtered extracts were dried under vacuum by rotary evaporator. The extracts were kept at 4 °C and reconstituted in 100% acetone to a concentration of 10 mg/mL. The stock solutions were subsequently diluted to the required concentrations for the respective assays. All the experiments were carried out in triplicate.

### 2.3. Phytochemical Screening

#### 2.3.1. Total Phenolic Content

The microplate total phenolic content (TPC) method, a modified Folin–Ciocalteu assay described by [25] and modified by [26], was used to determine total phenolic content. A 20 µL (10 mg/mL) extract was mixed with 20 µL Folin–Ciocalteu reagent, shaken, and after 5 min, combined with 200 µL of 7% sodium carbonate. The mixture was shaken and incubated in the dark for 120 min at room temperature. Absorbance was measured at 750 nm using a microplate reader (Thermo Scientific, Waltham, MA, USA), with a control reaction subtracted for accuracy. Gallic acid was used for calibration, and results (Table 3). were expressed as µg gallic acid equivalent/g dry extract.

#### 2.3.2. Estimation of Total Tannin Content

The microplate total tannin content, estimated using a modified Folin–Ciocalteu assay, was used to determine tannin content using the method described by [27]. Plant extracts were prepared at a concentration of 10 mg/mL. Briefly, 20 µL extract was mixed with 20 µL Folin–Ciocalteu reagent and shaken for 1 minute. After 5 min, 60 µL of 35% sodium carbonate and 120 µL of deionised water were added, followed by shaking. The mixture was incubated in the dark at room temperature for 30 min. Absorbance was measured at 750 nm using a microplate reader, with a control reaction subtracted for accuracy. The tannin content was reported as milligrams of gallic acid equivalent per gram of extract (mg GAE/g extract) (Table 3). Since the Folin–Ciocalteu reagent is not specific to tannins and can react with other phenolic and reducing compounds, the results were interpreted as an estimation of Folin–Ciocalteu-reactive tannin compounds.

#### 2.3.3. Total Flavonoid Content

The assay, performed in triplicate following [27], used a 96-well plate method. Each well contained 100 µL of distilled water, 10 µL of 50 g/L sodium nitrite (NaNO_2_), and 25 µL of either a standard or sample solution (70% acetone). After 5 minutes, 15 µL of 10% aluminium chloride (AlCl_3_) (dissolved in distilled water) was added, followed by 50 µL of 1 mol/L NaOH and 50 µL of distilled water after 6 min. The plate was shaken for 30 seconds before absorbance was measured at 510 nm using a microplate reader (Thermo Scientific, Waltham, MA, USA). Quercetin was used as a standard, and results were expressed as mg quercetin equivalent/g extract, with an acetone reagent blank for reference (Table 3).

### 2.4. Analysis of Plant Extracts Using High-Performance Thin-Layer Chromatography (HPTLC)

HPTLC was performed for preliminary qualitative chemical profiling and fingerprinting of the plant extracts, rather than for compound identification or quantification. A 2 μL aliquot of methanol, dichloromethane (DCM), water, and petroleum ether prepared at a concentration of 10 mg/mL was applied onto a silica gel 60 F254 glass plate (20 × 10 cm) (Merck Ltd., Darmstadt, Germany). Three distinct solvent systems were utilised as mobile phases—benzene/ethanol/ammonium hydroxide (BEA), chloroform/ethyl acetate/formic acid (CEF), and ethyl acetate/methanol/water (EMW)—to reflect the varying polarities of the phytochemicals being analysed [28]. The optimal solvent system for development consisted of chloroform, ethyl acetate, methanol, and formic acid at a ratio of 90:10:20:1. To prepare the chamber, 25 mL of the solvent was used for saturation for 20 min at 33% relative humidity and a temperature of 23 ± 2 °C. Once saturation was complete, 10 mL of the mobile phase was introduced, allowing migration over a 70 mm distance. After development, the plate was automatically dried in the chamber for six min before being examined under UV light at 254 nm and 366 nm, as well as under white light using an automated visualiser (CAMAG, Muttenz, Switzerland). For compound visualisation, the plate was immersed in a vanillin solution (prepared by dissolving 0.1 g vanillin in 28 mL methanol and 1 mL sulfuric acid) for a few seconds at a uniform vertical speed level of 3 using a chromatogram immersion device. This was followed by heating the plate at 100 °C for three min on a plate heater. Finally, the plate was allowed to cool at room temperature for three min before analysis [29].

### 2.5. Antibacterial Activity of Plant Extract

#### 2.5.1. Bacteria Used in the Study

Antibacterial, synergistic, and anti-biofilm activities of the plant extracts were evaluated against four bacterial pathogens commonly associated with oral infections: *Porphyromonas gingivalis* (ATCC 33277) (Gram-negative), *Pseudomonas aeruginosa* (ATCC 27853), *Streptococcus mutans* (ATCC 25175), *Enterococcus faecalis* (ATCC 19433), and *Staphylococcus aureus* (ATCC 29213) (Gram-positive). All bacterial strains were cultured at 37 °C. Gram-negative and Gram-positive bacteria were grown in Mueller–Hinton (MH) broth under appropriate conditions prior to testing.

#### 2.5.2. Broth Micro-Dilution Assay

The broth micro-dilution assay was used to determine antibacterial activity [30]. The plant extracts (10,000 µg/mL) were serially diluted 2-fold to a final volume of 100 μL in a 96-well microtitre plate to achieve a concentration of 20–2500 µg/mL using 100 µL of sterile media. *S. mutans* (2.7 × 10^6^ CFU/mL), *P. gingivalis* (2.05 × 10^6^ CFU/mL), *E. faecalis* (2.4 × 10^6^ CFU/mL), *P. aeruginosa* (2.1 × 10^6^ CFU/mL) and *S. aureus* (2.1 × 10^6^ CFU/mL) were cultured overnight in 100 μL wells. The positive control was gentamicin (100 μl of 0.1 µg/mL), whereas the negative control was the media and acetone. The 96-well plates were incubated at 37 °C for 24 h. After incubation, 40 μL of 0.2 mg/mL *p*-iodonitrotetrazolium chloride (INT) (Sigma-Aldrich, St. Louis, MO, USA) was added to each well and incubated for 30 minutes. The MIC was recorded as the lowest concentration of the extract that showed no visible colour change (pink to reddish-purple) after addition of INT, indicating inhibition of microbial growth. The growth control exhibited a pink to reddish-purple colour change, confirming microbial viability.

#### 2.5.3. Evaluation of Combinational Effects of Plant Extracts 

The study evaluated antibacterial effects of plant extract combinations using the micro-dilution assay. Methanolic extracts of the five medicinal plants were selected for the combination studies based on their superior antibacterial activity. Each plant extract was prepared at 10.000 µg/mL and mixed in a 1:1 (*v*/*v*) ratio, resulting in a final concentration of 5 mg/mL for each extract (10.000 total extract concentration). The fractional inhibitory concentration (FIC) was determined [30] and the FIC index (FICI) values were interpreted according to the criteria presented in Table 2.

The FIC for each extract was calculated as

$$\mathrm{FIC}=\frac{\mathrm{MIC}\left(\mathrm{combination a},b\right)}{\mathrm{MIC a}}$$

The total FIC index was then obtained by summing individual FIC values:

∑FIC = FIC_A_ + FIC_B_

FIC_A_ represents the FIC of the first extract in the combination, while FIC_B_ corresponds to the second extract (Table 2), where MIC represents the minimum inhibitory concentration.

**Table 2:** Interpretations of combinational effects of different plant extracts [31].

FIC Index Value	Outcome of the Combination
∑ FIC ≤ 0.5	Synergistic
∑ FIC > 0.5–1.00	Additive
∑ FIC > 1.00 ≤ 4.00	Indifferent
∑ FIC > 4.00	Antagonistic

Key: ∑ FIC: Fractional inhibitory index.

### 2.6. Anti-Biofilm Screening Assay

The antibiofilm activity of the extracts was evaluated using the crystal violet assay to assess biofilm prevention, inhibition of initial cell attachment, and eradication of preformed biofilms, focusing on assessing the efficacy of the extracts in inhibiting the growth of biofilms or eradicating pre-existing biofilms [32]. The bacterial strains used were *S. mutans*, *S. aureus*, *E. faecalis*, *P. aeruginosa* and *P. gingivalis*.

#### 2.6.1. Common Biofilm Assay Procedure

Bacterial cultures were prepared by inoculating the respective bacterial strains into nutrient broth and incubating them under the appropriate growth conditions. The resulting bacterial suspensions were standardised to an absorbance_600_ of 0.02 using a UV/VIS spectrophotometer (Genesys 10S UV-VIS, Thermo Scientific, Waltham, MA, USA), corresponding to viable counts of 2.7 × 10^6^ CFU/mL for *S. mutans*, 2.05 × 10^6^ CFU/mL for *P. gingivalis*, 2.4 × 10^6^ CFU/mL for *E. faecalis*, 2.1 × 10^6^ CFU/mL for *P. aeruginosa* and 2.1 × 10^6^ CFU/mL for *S. aureus*.

The standardised suspensions were used for the prevention of biofilm formation, inhibition of initial cell attachment and biofilm eradication assays. These concentrations were calculated individually for each plant extract against each microorganism based on the respective MIC values presented in Table 4. Gentamicin (0.1 µg/mL) was used as the positive control, while untreated bacterial cultures and sterile medium served as negative controls. The final volumes and incubation conditions for each assay are described in Section 2.6.2, Section 2.6.3 and Section 2.6.4.

Following the respective treatment and incubation periods, the contents of the wells were removed, and the wells were rinsed three times with sterile distilled water to remove non-adherent bacterial cells. The plates were oven-dried at 60 °C for 45 min. Each well was then stained with 100 µL of 0.1% crystal violet solution and incubated at room temperature for 15 min. Excess crystal violet was removed, and the wells were washed three times with sterile distilled water. The retained crystal violet was solubilised by adding 100 µL of methanol to each well. The solubilised stain was transferred to a new microtiter plate, and absorbance was measured at 590 nm using a microplate reader [33].

The percentage inhibition of biofilm biomass was calculated using the following equation:

$$\mathrm{Biofilm}\;\mathrm{inhibition}\;(\%)\;=\;\frac{\mathrm{absorbanceUT}-\mathrm{absorbanceS}}{\mathrm{absorbanceUT}}\times 100$$

where absorbanceUT represents the absorbance of the untreated control and absorbanceS represents the absorbance of the extract-treated sample. The MBIC_50_ was subsequently determined from the concentration–response data using nonlinear regression analysis in GraphPad Prism version 9.0. MBIC_50_ was defined as the concentration of the extract required to inhibit 50% of biofilm formation relative to the untreated control, providing an estimate of the concentration needed to achieve a 50% reduction in biofilm biomass.

#### 2.6.2. Prevention of Biofilm Formation

The prevention assay evaluated the ability of the plant extracts to prevent biofilm formation during bacterial growth. Nutrient broth (100 µL) was added to each well of a microtiter plate, followed by 100 µL of the standardised bacterial suspension. Subsequently, 100 µL of the plant extract was added. The extracts were present during the biofilm development period to assess their ability to prevent biofilm formation, and the plates were incubated at 37 °C for 24 h [30]. Following incubation, the biofilm biomass was quantified using the crystal violet staining and absorbance measurement procedure described in Section 2.6.1.

#### 2.6.3. Inhibition of Initial Cell Attachment

The initial attachment assay evaluated the ability of the plant extracts to inhibit the early attachment of bacterial cells to the surface. A volume of 100 µL of the standardised bacterial suspension was added to each well and incubated at 37 °C for 4 h without agitation to allow initial bacterial attachment, based on the protocol described by [32]. After the 4 h attachment period, 100 µL of the plant extract was added. The plates were subsequently incubated at 37 °C for a further 24 h without agitation [33]. The remaining attached biomass was quantified using the crystal violet staining and absorbance measurement procedure described in Section 2.6.1.

#### 2.6.4. Biofilm Eradication Assay

The biofilm eradication assay evaluated the ability of the plant extracts to reduce an already established biofilm. A volume of 100 µL of the standardised bacterial suspension was added to each well and incubated at 37 °C for 48 h to allow an established biofilm to form before treatment. Following incubation, 100 µL of the plant extract was added. The plates were then incubated for an additional 24 h at 37 °C [33]. The remaining biofilm biomass following treatment was quantified using the crystal violet staining and absorbance measurement procedure described in Section 2.6.1.

### 2.7. Statistical Analysis

Analysis of the polyphenolic content data was performed using Analysis of Variance (ANOVA) with SPSS software (IBM, Armonk, New York, USA), version 29.0.0.0 (241). Results were expressed as means ± standard deviation of triplicate determinations. Statistical analysis was performed by Graph Prism version 9 with a two-way analysis of variance followed by Tukey’s multiple comparison test at a significance level of *p* ≤ 0.05. Principal Component Analysis (PCA) was performed using MetaboAnalyst version 6.0, and a biplot was generated to visualise similarities and differences among plant extracts based on their total phenolic, flavonoid, and tannin contents.

## 3. Results

### 3.1. High-Performance Thin-Layer Chromatography (HPTLC)

**Figure 2:**
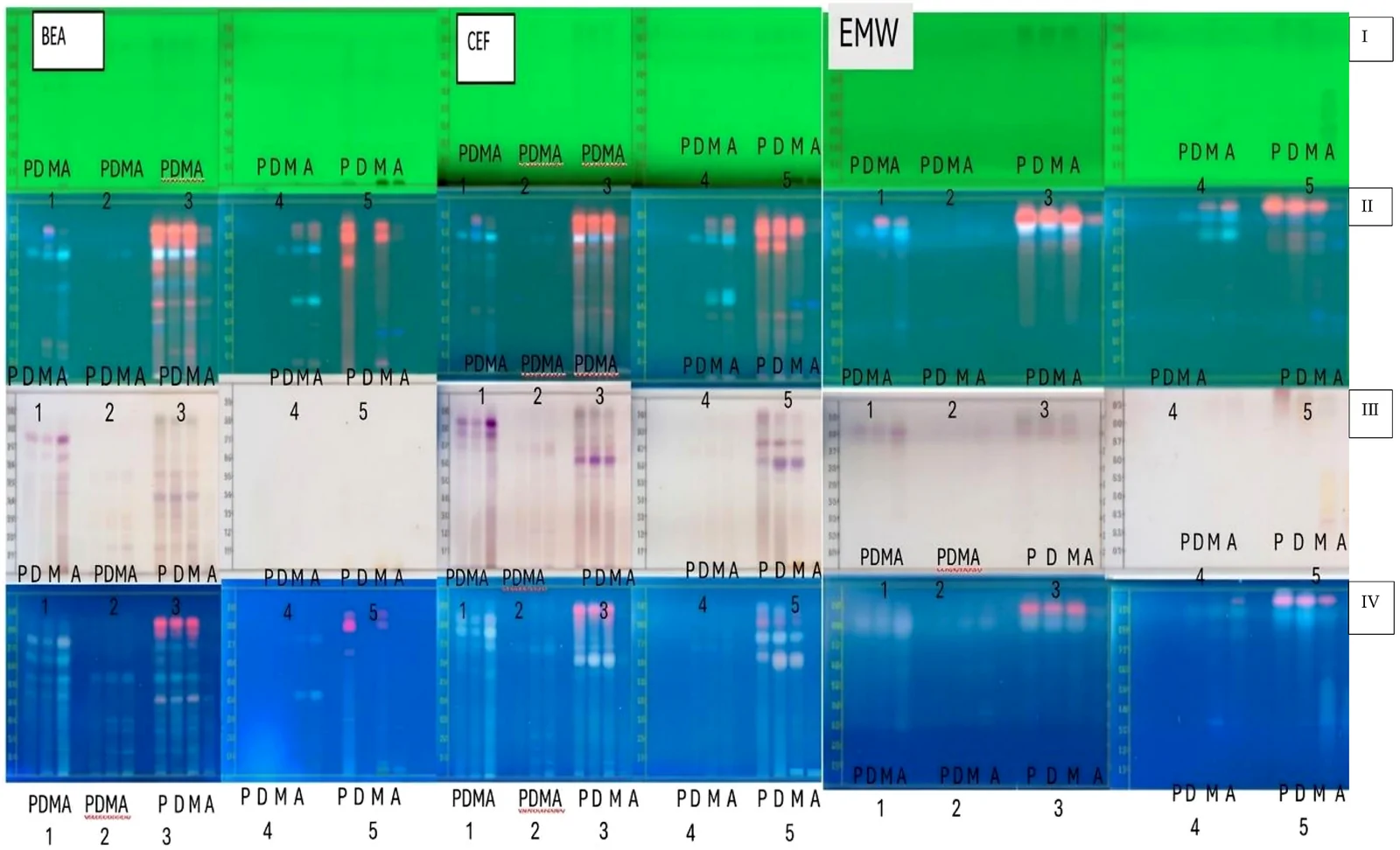
Phytochemical (plate **I**–**III**) bands are shown in the TLC chromatogram of the five plants. The BEA, CEF, and EMW mobile phases were used to produce the chromatograms. Chromatograms were visualised under UV light at 254 nm (**I**) and 365 nm (**II**). After derivatisation with vanillin–sulphuric-acid reagent, chromatograms were visualised under white light (**III**) and under 365 nm UV light after derivatisation (**IV**). Key: (P) Petroleum ether, (D) dichloromethane, (M) methanol, (A) water/aqueous. 1 *Kleinia longiflora*; 2 *Drimia elata* Jacq.; 3 *Sesamum senecioides*; 4 *Aloe greatheadii*; 5 *Euclea undulata*.

High-performance thin-layer chromatography (HPTLC) was employed for phytochemical screening, offering a precise and cost-effective method [34]. Extracts from *S. senecioides*, *E. undulata*, *K. longiflora*, *A. greatheadii*, and *D. elata*, prepared using various solvents, were analysed under UV light at 254 nm and 366 nm, with non-fluorescent compounds detected using a vanillin–sulfuric-acid reagent (Figure 2). After derivatisation with vanillin–sulphuric acid reagent, chromatograms were visualised under white light (III) and under 365 nm UV light after derivatisation (IV) (Figure 2). The retention factor (Rf) values varied according to the solvent system BEA, CEF, and EMW, indicating differences in phytochemical polarity.

The retention factor (Rf) values varied according to the solvent system BEA, CEF, and EMW, indicating differences in phytochemical polarity. At 254 nm, the CEF and EMW systems revealed a higher number of compounds, with phenolic derivatives and conjugated double-bond-containing compounds appearing as dark bands [29]. At 366 nm, the BEA system exhibited intense blue fluorescence, characteristic of alkaloids, flavonoids, and terpene alcohols [35]. Vanillin–sulfuric-acid staining further confirmed these findings, showing consistent results at 365 nm [33]. Among the three solvent systems, BEA demonstrated the highest resolution, while both EMW and BEA effectively separated polar and non-polar compounds. The EMW system efficiently extracted phenols and flavonoids, whereas BEA facilitated the separation of alkaloids and nitrogen-containing compounds, producing distinct bands.

### 3.2. Quantification of Total Phenolic, Flavonoid, and Tannin Content

**Figure 3:**
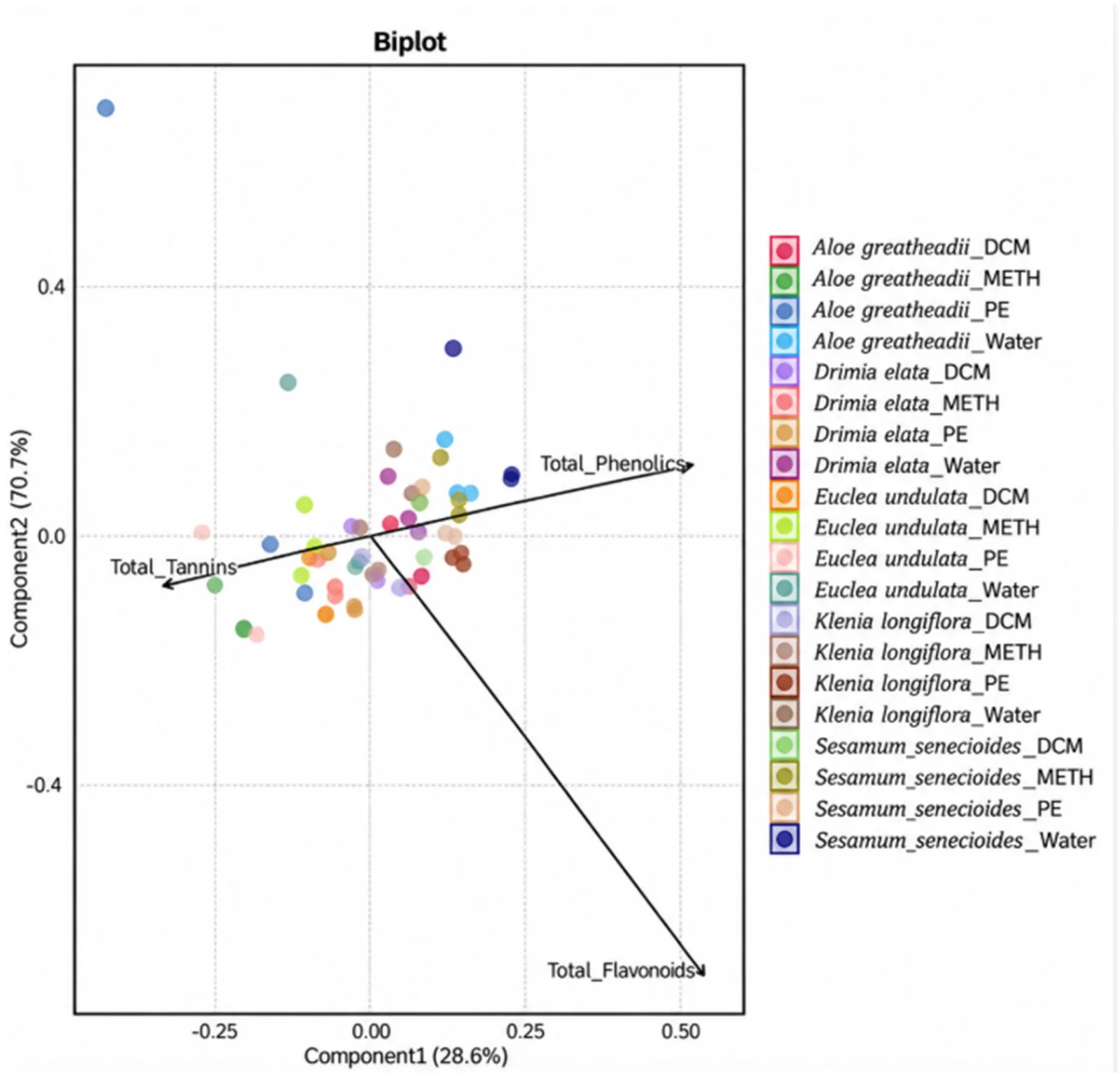
PCA biplot showing the phytochemical profiles of plant extracts based on total phenolic, flavonoid, and tannin contents. PC1 and PC2 explained 99.3% of the total variance. **Keys**: PE: Petroleum ether; DCM: dichloromethane; METH: methanol.

The phytochemical analysis of five plant species revealed that *E. undulata* possessed the highest concentrations of phenolics, flavonoids, and tannins across various solvent extracts. Specifically, its water and methanol extracts contain phenolic contents of 377.63 ± 2.25 mg GAE/g and 269.84 ± 2.76 mg GAE/g, respectively. Additionally, the PE and DCM extracts exhibited flavonoid levels of 31.26 ± 2.76 mg GAE/g and 25.98 ± 2.54 mg GAE/g, respectively, while the PE extract also showed a tannin concentration of 2400.74 ± 8.31 mg GAE/g (Table 3). The PCA further supported these variations (Figure 3), with PC1 and PC2 explaining 28.6% and 70.7% of the total variation, respectively (99.3% cumulative variance). Phenolics were mainly associated with the positive PC1 direction, particularly the *E. undulata* water extract. Flavonoids were associated with the positive PC1 and negative PC2 directions, particularly in *E. undulata* PE and DCM, *K. longiflora* PE, and *S. senecioides* METH extracts. Tannins contributed mainly to the negative PC1 direction, particularly in *E. undulata* PE and *S. senecioides* METH extracts. Overall, the PCA confirmed that plant species and extraction solvent influenced the phytochemical profiles of the extracts.

### 3.3. Antibacterial Activity of Medicinal Plant Extracts

The extracts exhibited varying antibacterial activity against the tested bacterial strains. *Euclea undulata* and *Sesamum senecioides* showed the strongest activity, particularly their methanolic extracts, with MIC values as low as 0.15 µg/mL against *S. mutans* and *P. gingivalis*. *Aloe greatheadii* also showed notable activity, particularly against *P. aeruginosa* and *P. gingivalis*, with MIC values of 0.31 µg/mL. In contrast, *K.longiflora* and *D. elata* generally showed weaker activity, with most MIC values ranging from 1.25 to >2.5 µg/mL. The methanolic extract of *K. longiflora* exhibited MIC values of 0.63–2.5 µg/mL, whereas *D. elata* showed values of 0.63–>2.5 µg/mL. Gentamicin, used as the positive control, exhibited substantially lower MIC values of 0.0031–0.16 µg/mL across the tested bacteria, confirming its strong antibacterial activity. Overall, the plant extracts exhibited antibacterial activity, although their potency varied considerably among plant species, extraction solvents, and bacterial strains.

**Table 4:** Minimum inhibitory concentration (MIC) of various plant extracts against four bacterial strains.

Plants	Extract	*P. aeruginosa* (µg/mL)	*E. faecalis* (µg/mL)	*S. aureus* (µg/mL)	*S. mutans* (µg/mL)	*P. gingivalis* (µg/mL)
*Sesamum senecioides*	PE	>2.5	>2.5	>2.5	>2.5	>2.5
	DCM	2.5	>2.5	1.25	1.25	1.25
	METH	0.63	0.63	0.31	0.31	0.15
	Water	0.63	>2.5	>2.5	>2.5	>2.5
*Kleinia longiflora*	PE	>2.5	>2.5	>2.5	>2.5	>2.5
	DCM	>2.5	>2.5	>2.5	1.25	>2.5
	METH	2.5	2.5	0.63	0.63	1.25
	Water	>2.5	>2.5	>2.5	>2.5	>2.5
*Drimia elata*	PE	>2.5	>2.5	>2.5	>2.5	>2.5
	DCM	>2.5	>2.5	>2.5	>2.5	>2.5
	METH	2.5	>2.5	2.5	0.63	0.63
	Water	>2.5	>2.5	>2.5	>2.5	>2.5
*Euclea Undulata*	PE	2.5	1.25	2.5	2.5	>2.5
	DCM	>2.5	0.63	1.25	1.25	1.25
	METH	0.63	1.25	0.63	0.15	0.15
	Water	0.63	1.25	0.63	2.5	1.25
*Aloe greatheadii*	PE	>2.5	>2.5	>2.5	>2.5	2.5
	DCM	>2.5	>2.5	>2.5	1.25	>2.5
	METH	0.31	2.5	2.5	0.63	0.31
	Water	>2.5	>2.5	>2.5	>2.5	>2.5
Gentamicin		0.0031	0.16	0.0078	0.0031	0.0031

Note: MIC values of ≤1 µg/mL indicate good antibacterial activity, whereas values >1 µg/mL indicate weaker antibacterial activity. The lower the MIC, the stronger the antibacterial activity, because a smaller concentration of the extract is required to inhibit bacterial growth. Keys: PE: Petroleum ether, DCM: dichloromethane, METH: methanol.

### 3.4. Combinational Effects of Plant Extracts

The study assessed the antibacterial activity of plant extracts in combination against *S. aureus*, *S. mutans*, *E. faecalis*, *P. aeruginosa* and *P. gingivalis* using the MIC and fractional inhibitory concentration (FIC) indices, respectively. The findings revealed synergistic, indifferent, additive, and antagonistic effects. Most tested plant extract combinations exhibited indifferent effects against *S. aureus*, indicating no significant change in antibacterial activity compared to individual extracts. However, combinations involving *E. undulata* (*A. greatheadii* + *E. undulata* and *E. undulata* + *K. longiflora*) displayed an additive effect, suggesting a mild enhancement in antibacterial efficacy (Table 5). This effect may be associated with the combined activity of phytochemical constituents present in the extracts. *E. undulata* contains phenolic and flavonoid compounds that may contribute to antibacterial activity through effects on bacterial cell membranes and cellular functions. However, the specific compounds responsible for the observed additive effect were not determined in this study. The results for *S. mutans* (Table 6) exhibited both indifferent and antagonistic interactions. Notably, combinations involving *E. undulata* with *K. longiflora*, *D. elata*, and *S. senecioides* showed antagonistic effects with high FIC values. All plant combinations exhibited antagonistic effects against *P. gingivalis*, indicating that the combined extracts diminished antibacterial activity rather than enhancing it (Table 7). The present study also demonstrated that combinations of these extracts exhibited synergistic effects against various pathogens, including *P. aeruginosa* (Table 8). This finding indicates that the combined extracts had a greater antibacterial effect against *P. aeruginosa* than when the extracts were used individually. The combination of *E. undulata* and *K. longiflora* against *P. aeruginosa* exhibited a synergistic effect. Most of the combinations observed in Table 9 are neither synergistic nor additive against *E. faecalis*.

### 3.5. Antibiofilm Activity

**Figure 4:**
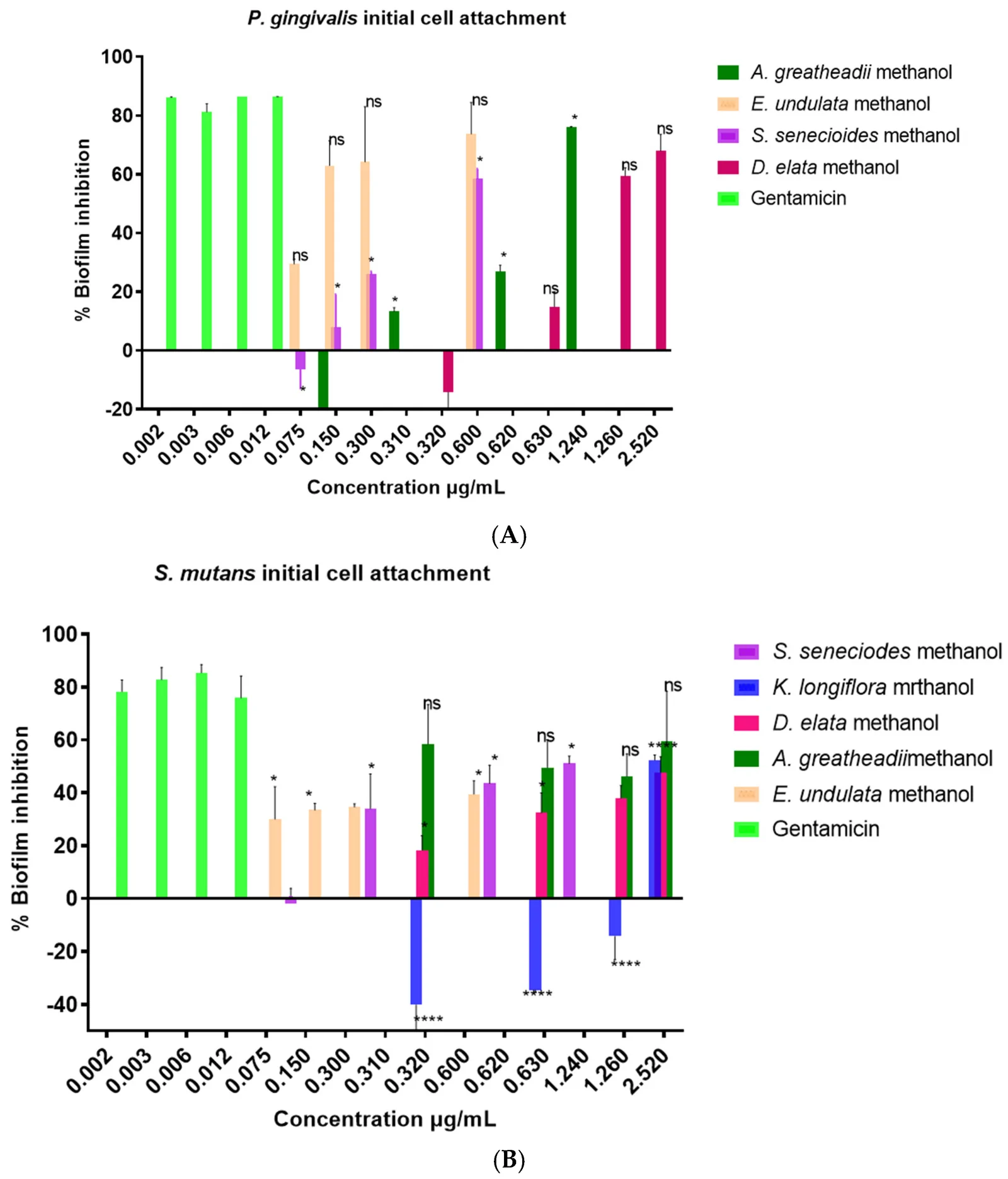

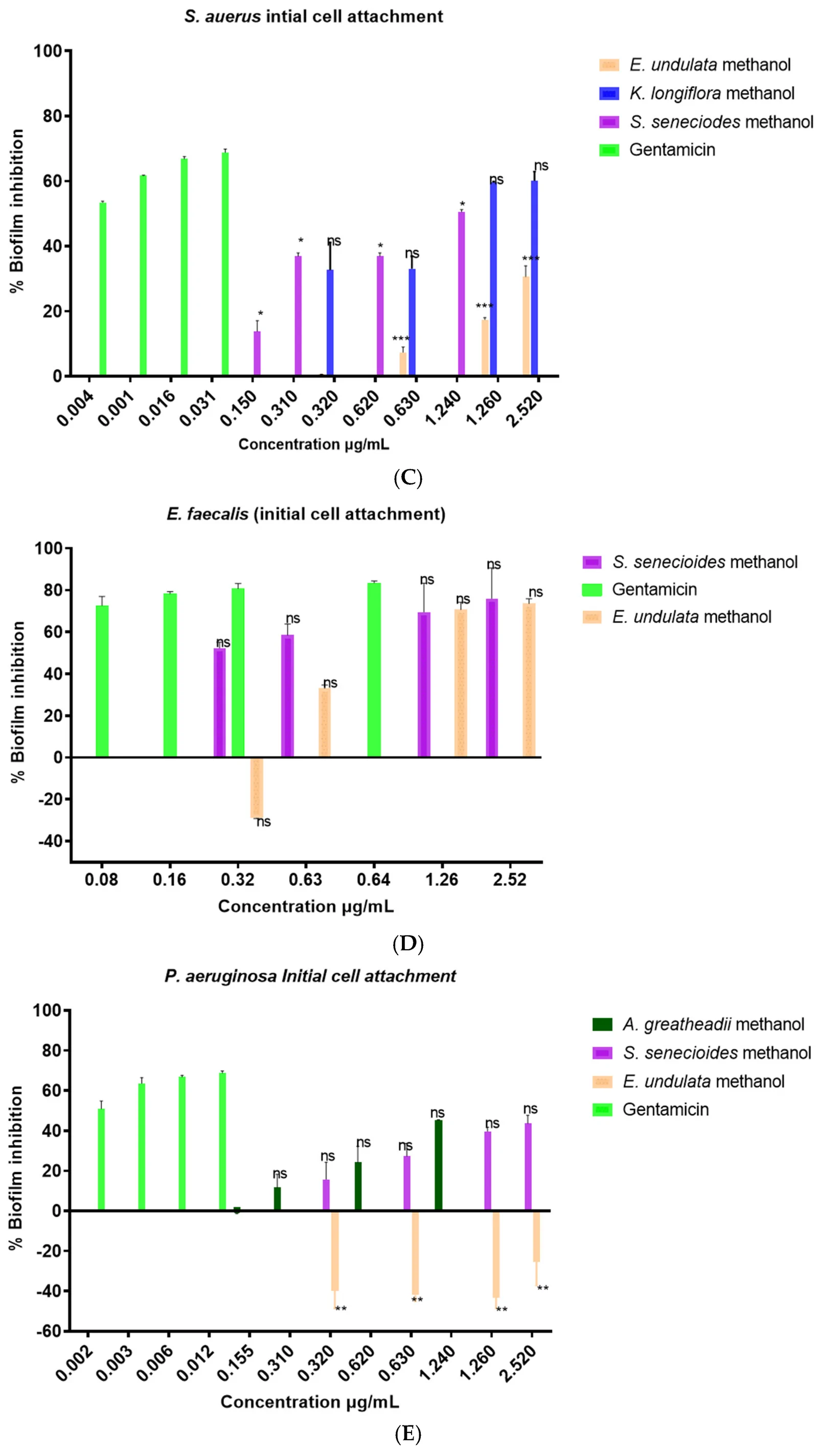
Inhibition of cell attachment of *P. gingivalis* (**A**), *S. mutans* (**B**), *S. aureus* (**C**), *E. faecalis* (**D**), and *P. aeruginosa* (**E**). ns: non-significance (*p* > 0.05); (∗): *p* < 0.05; (∗∗): *p* < 0.01; (∗∗∗): *p* < 0.001; (∗∗∗∗): *p* < 0.0001.

**Figure 5:**
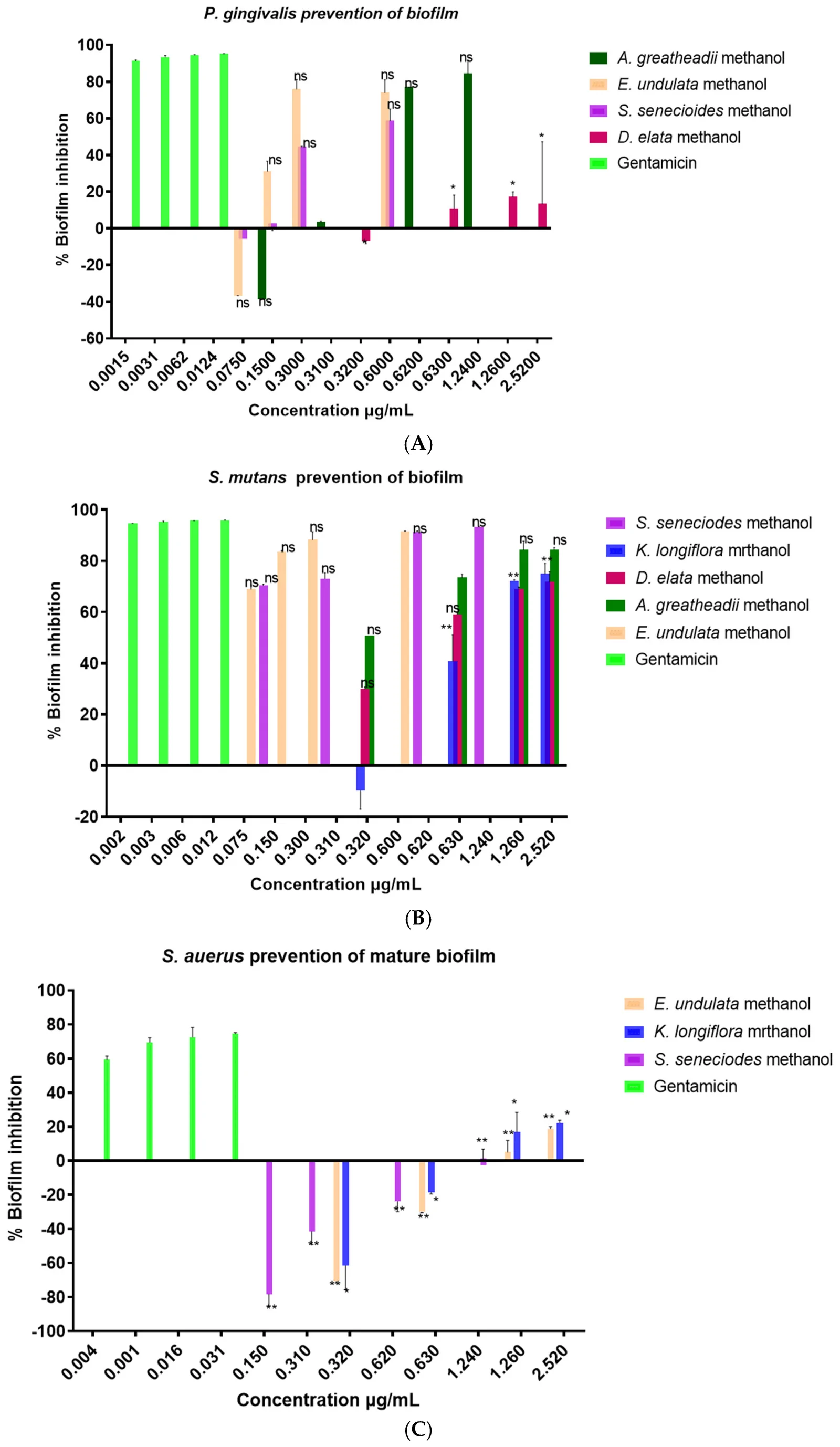

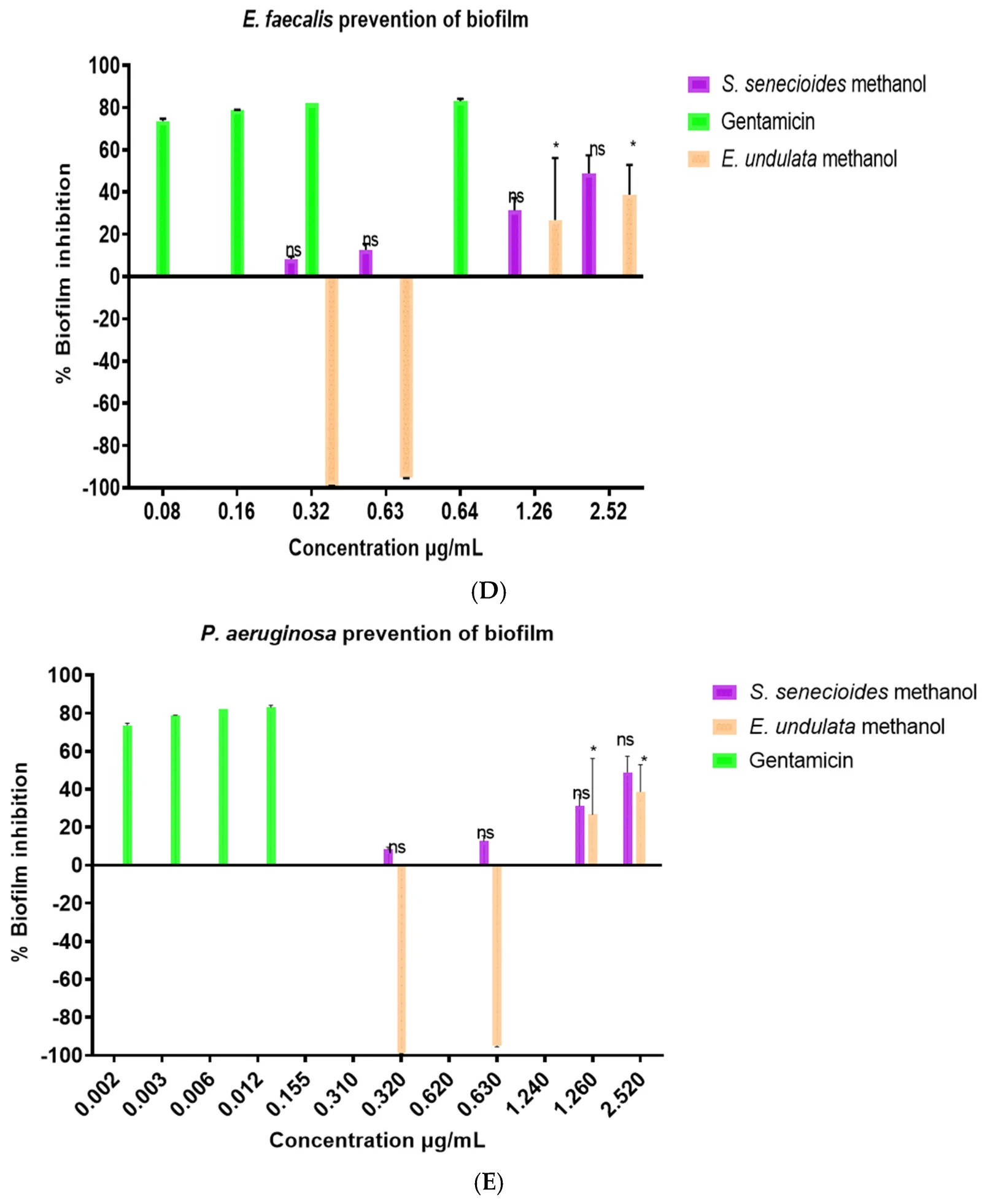
Prevention of biofilms of *P. gingivalis* (**A**), *S. mutans* (**B**), *S. aureus* (**C**), *E. faecalis* (**D**), and *P. aeruginosa* (**E**). ns: non-significance (*p* > 0.05); (∗): *p* < 0.05; (∗∗): *p* < 0.01.

The methanol extracts demonstrated organism-, concentration-, and biofilm-stage-dependent antibiofilm activity (Figure 4 and Figure 5). In this study, >50% inhibition was considered good activity, <50% indicated limited activity, and negative values indicated a pro-biofilm effect.

**Figure 6:**
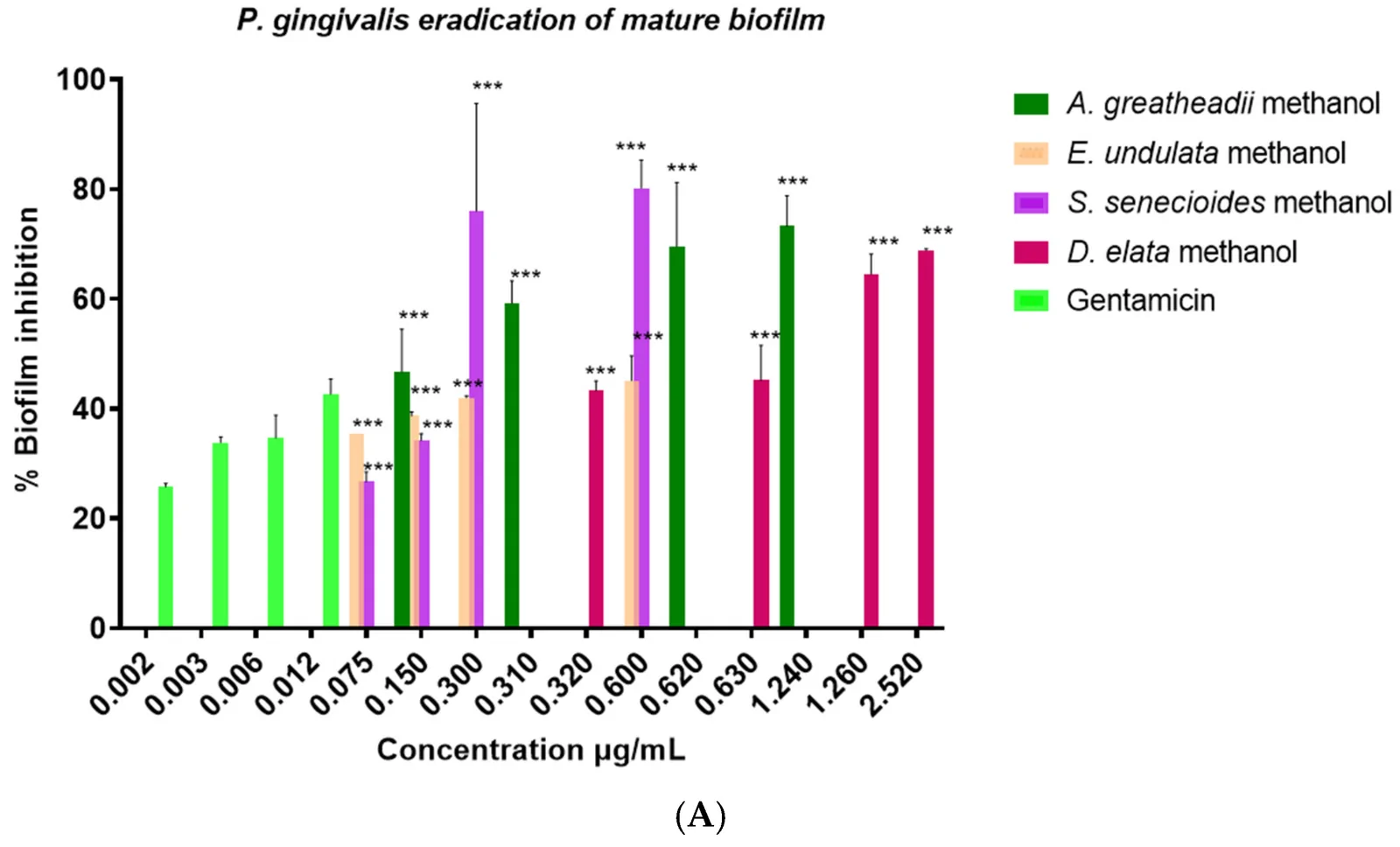

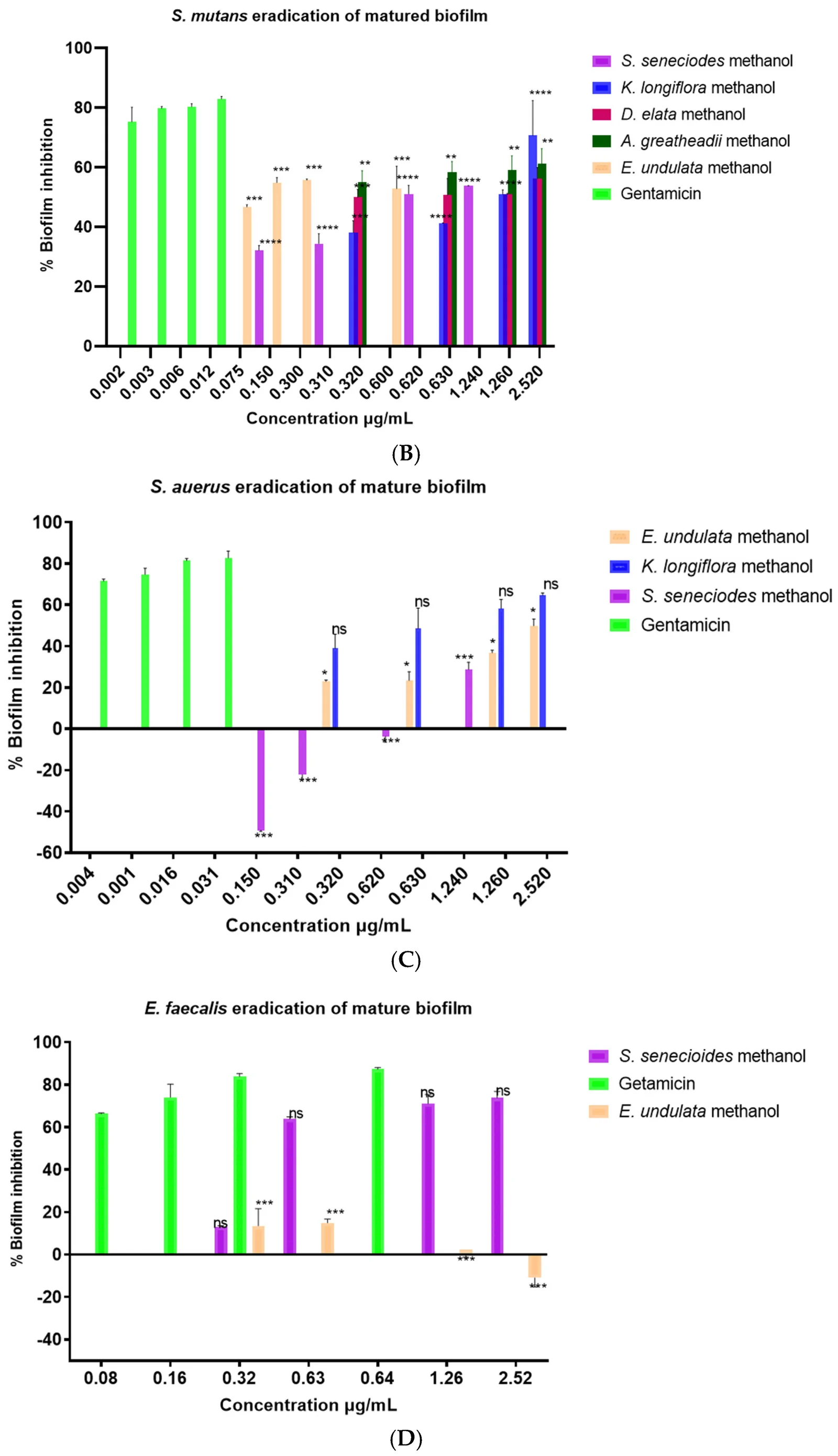

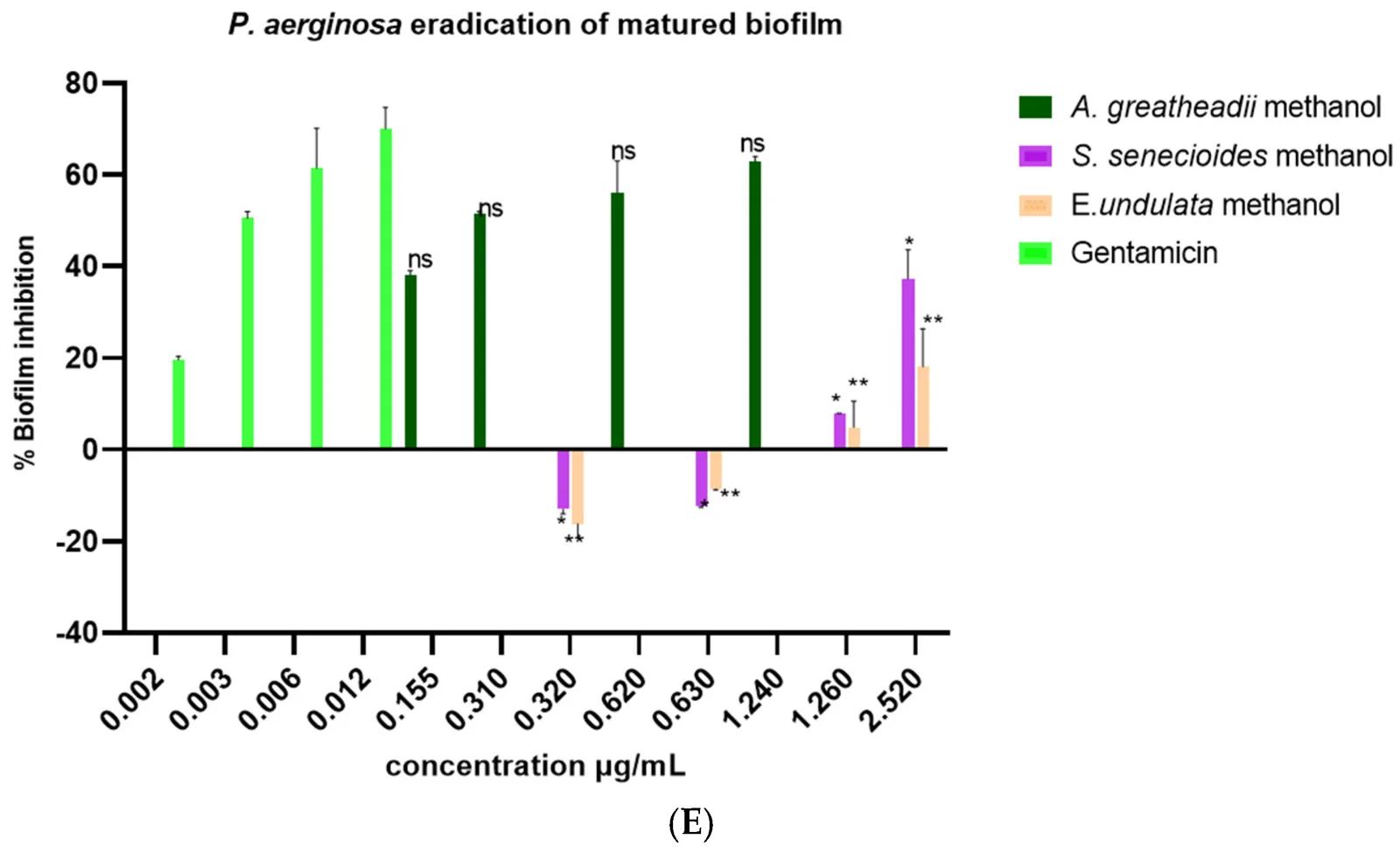
Eradication of mature biofilms of *P. gingivalis* (**A**), *S. mutans* (**B**), *S. aureus* (**C**), *E. faecalis* (**D**), and *P. aeruginosa* (**E**). ns: non-significance (*p* > 0.05); (∗): *p*< 0.05; (∗∗): *p* < 0.01; (∗∗∗): *p* < 0.001; (∗∗∗∗): *p* < 0.000.

During initial cell attachment, *A. greatheadii* showed the strongest inhibition against *P. gingivalis*, reaching approximately 75% at 0.60 µg/mL, while *S. senecioides* exhibited approximately 75% inhibition against *E. faecalis* at 2.52 µg/mL (Figure 4). *Klenia longiflora* also exhibited good activity against *S. aureus* at 2.52 µg/mL and 1.26 µg/mL ~60% inhibition. However, *E. undulata* (0.320–2.520 µg/mL) against *P. aueruginosa* and *K. longiflora* (0.63 and 1.26 µg/mL) exhibited negative inhibition values at certain concentrations, indicating pro-biofilm effects (Figure 4C). For biofilm prevention, *A. greatheadii* exhibited the strongest activity against *P. gingivalis*, with approximately 90–95% inhibition, and against *P. aeruginosa*, reaching approximately 100% inhibition at 1.24 µg/mL (Figure 5). Several extracts also showed good inhibition of *S. mutans*, particularly at higher concentrations (0.63, 1.260 and 2.50 µg/mL). In contrast, *E. undulata* exhibited negative inhibition against *P. aeruginosa* (0.32 and 0.63 µg/mL) and *E. faecalis* at some concentrations (0.32 µg/mL and 0.63 µg/mL), indicating pro-biofilm activity. In the eradication of established biofilms, *A. greatheadii* exhibited strong activity against *S. mutans* (~80–85% at 0.63–2.52µg/mL) and *P. aeruginosa* (~60–70% at 0.62 and 1.24 µg/mL). *S. senecioides* demonstrated good activity against *P. gingivalis* (~80% at 0.62 and 1.24 µg/mL) and *E. faecalis* (~75% at 2.52 µg/mL), while *K. longiflora* exhibited good activity against *S. aureus* at 2.52 µg/mL and ~55–60% at 0.63 µg/mL (Figure 6).

Overall, *A. greatheadii* exhibited the most consistent antibiofilm activity, particularly against *P. gingivalis*, *S. mutans* and *P. aeruginosa*, while *S. senecioides* was particularly effective against *P. gingivalis* and *E. faecalis*. The occurrence of negative values for some extract–organism combinations highlights that antibiofilm activity was species-, concentration- and biofilm-stage-dependent (Figure 4, Figure 5 and Figure 6).

The MBIC_50_ values showed clear differences in antibiofilm potency among the plant extracts and bacterial strains, with lower MBIC_50_ values indicating greater activity (Table 10). During biofilm prevention, *E. undulata* exhibited the strongest activity against *S. mutans* (0.004 µg/mL), while *D. elata* was most active against *P. aeruginosa* (0.05 µg/mL). For initial cell attachment, *D. elata* exhibited the lowest MBIC_50_ against *S. mutans* (0.001 mg/mL), followed by *S. senecioides* against *S. aureus* (0.002 µg/mL). In the eradication of established biofilms, *A. greatheadii* exhibited the highest potency against *S. mutans* (7.91 × 10^−6^ µg/mL) and *P. aeruginosa* (0.006 µg/mL), whereas *S. senecioides* was most potent against *E. faecalis* (0.06 µg/mL). Overall, the results exhibited strong but strain- and biofilm-stage-dependent antibiofilm activity, with *A. greatheadii* showing particularly notable potency against established biofilms.

## 4. Discussion

High-performance thin-layer chromatography (HPTLC) was employed for qualitative phytochemical screening and profiling of the plant extract [29]. Extracts from *S. senecioides*, *E. undulata*, *K. longiflora*, *A. greatheadii*, and *D. elata*, prepared using various solvents, were analysed under UV light at 254 nm and 366 nm, with non-fluorescent compounds detected using a vanillin-sulfuric acid reagent. After derivatisation with vanillin–sulphuric-acid reagent, chromatograms were visualised under white light (III) and under 365 nm UV light after derivatisation (IV) (Figure 2). The retention factor (Rf) values varied according to the solvent systems BEA, CEF, and EMW, indicating differences in phytochemical polarity.

The HPTLC fingerprints obtained using the BEA, CEF, and EMW solvent systems revealed clear differences in the phytochemical composition of the five medicinal plants, as evidenced by variations in band number, fluorescence, and colour intensity under UV light. These qualitative chromatographic profiles complemented the quantitative phytochemical findings (Table 3), demonstrating a relationship between metabolite abundance and HPTLC band intensity. Extracts of *E. undulata* showed the most prominent fluorescent bands, particularly in the EMW and CEF solvent systems, which correlated with its high phenolic, flavonoid, and tannin contents. This is consistent with [15] who reported considerable phytochemical diversity within the genus *Euclea*, including flavonoids, terpenoids, tannins and naphthoquinones.

The aqueous and methanol extracts of *E. undulata* exhibited phenolic concentrations of 377.63 ± 2.25 mg GAE/g and 269.84 ± 2.76 mg GAE/g, respectively, while the PE extract recorded the highest tannin content (2400.74 ± 8.31 mg GAE/g). Similar observations were reported for *Euclea schimperi*, where leaf extracts contained high phenolic levels with comparatively lower flavonoid concentrations [36]. Likewise, studies on *Cadaba rotundifolia* demonstrated that extraction efficiency and phytochemical recovery are strongly influenced by solvent polarity [37]. The broader phytochemical literature on *Euclea* further supports the presence of diverse secondary metabolites across species, although their abundance varies according to species, plant part and extraction conditions.

The intense purple, blue, and pink bands visualised after vanillin–sulphuric-acid derivatisation further suggested the presence of flavonoids, terpenoids, and other phenolic-related compounds. Phenolic and flavonoid metabolites are known to fluoresce under UV light and commonly produce coloured bands following derivatisation, supporting the quantitative phytochemical results observed in this study [38,39]. Comparable colour and fluorescence patterns have also been reported in HPTLC-based phytochemical profiling of medicinal plants, demonstrating the usefulness of chromatographic fingerprints for qualitative assessment of complex plant extracts [38,39].

In contrast, *A. greatheadii*, *D. elata*, and *S. senecioides* aqueous extracts exhibited relatively low flavonoid concentrations ranging from 0.28 to 0.49 mg GAE/g, which corresponded with fewer and less intense bands on the HPTLC plates. Similar findings were reported by [40], who demonstrated that aqueous extracts of *Aloe vera* contained lower phenolic contents than methanol and ethanol extracts due to the limited ability of highly polar water to dissolve less polar phenolic compounds. Comparable observations in herbal infusions further emphasised the importance of solvent selection in enhancing phytochemical extraction efficiency [41].

The agreement between HPTLC fingerprinting and spectrophotometric quantification strengthens the reliability of the phytochemical profiling by confirming both the presence and relative abundance of secondary metabolites within the extracts. These metabolites may also contribute to the antibacterial and antibiofilm activities observed against oral pathogens. Phenolics, flavonoids, and tannins have been reported to inhibit microbial growth through membrane disruption, enzyme inhibition, interference with quorum sensing, and suppression of biofilm formation [42]. Therefore, the integration of HPTLC and quantitative phytochemical analyses provides evidence that the biological activities of these medicinal plants are closely associated with their chemically diverse metabolite composition.

The micro-dilution assay was used to determine the minimum inhibitory concentrations (MICs) of test extracts. The MIC values that were below 1.0 µg/mL were considered to have noteworthy antibacterial activity [43,44]. The methanol extracts of the studied medicinal plants demonstrated notable antibacterial activity against *S. mutans*, *P. gingivalis*, and *S. aureus*, with MICs ranging from 0.15 µg/mL to 0.63 µg/mL. *E. undulata* exhibited the most potent activity, with an MIC of 0.15 µg/mL against both *S. mutans* and *P. gingivalis*, and *S. senecioides*, with 0.15 µg/mL against *P. gingivalis*. Similarly, *S. senecioides* showed MICs of 0.31 µg/mL against *S. mutans* and *S. aureus* and 0.15 µg/mL against *P. gingivalis*. Other plants, including *K. longiflora*, *D. elata*, and *A. greatheadii*, also exhibited inhibitory effects, though at slightly higher MIC values (Table 4). The findings of the present study are consistent with research conducted by [43], which investigated the antibacterial properties of methanol extracts from 37 Korean medicinal plants. Their study identified *Camellia japonica* and *Thuja orientalis* as particularly effective, exhibiting MIC values of 0.5 µg/mL against *S. mutans* and *Candida albicans*. Additionally, these extracts significantly inhibited biofilm formation, highlighting their potential role in preventing dental infections [43]. Sudanese medicinal plants were also investigated, and it was found that methanol extracts of *Terminalia laxiflora* exhibited strong antibacterial activity against *P. gingivalis*, with an MIC of 0.25 µg/mL. Additionally, *Ambrosia maritima* and *Argemone mexicana* demonstrated inhibitory effects, emphasising the potential of medicinal plants in preventing oral pathogens.

Overall, the MIC values obtained in the present study are within the range of antibacterial activities reported for medicinal plant extracts against oral and other pathogenic bacteria. Differences in MIC values between studies may be related to variations in plant species, extraction solvents, plant material, geographical origin, bacterial strains and experimental conditions. Previous phytochemical investigations have reported several classes of bioactive constituents in medicinal plants, including phenolics, flavonoids, alkaloids, tannins and terpenoids, which may contribute to their antimicrobial properties [43,44]. However, these compounds were not identified or quantified in the present study; therefore, their direct contribution to the antibacterial activity observed here cannot be confirmed. The compounds discussed in relation to the present findings are based on previously published studies and should not be interpreted as compounds identified in the current extracts.

The study assessed the antibacterial activity of plant extracts in combination against *S. aureus*, *S. mutans*, *E. faecalis*, *P. aeruginosa* and *P. gingivalis* using the MIC and fractional inhibitory concentration (FIC) indices, respectively. The findings revealed indifferent, additive, and antagonistic effects (Table 2). The combinations produced different interaction patterns, including indifferent, additive, synergistic and antagonistic effects (Table 5, Table 6, Table 7, Table 8 and Table 9), demonstrating that the interaction depended on both the plant extract combination and the bacterial strain.

Most tested plant extract combinations exhibited indifferent effects against *S. aureus*, indicating no significant change in antibacterial activity compared to individual extracts. However, combinations involving *E. undulata* (*A. greatheadii* + *E. undulata* and *E. undulata* + *K. longiflora*, exhibited an additive effect, suggesting a mild enhancement in antibacterial efficacy (Table 5). These findings indicate that additive effects were limited to selected combinations rather than being a general feature of extract combinations. This may be due to the synergistic interaction of their phytochemical compounds. *E. undulata is* rich in phenolic and flavonoid compounds, which can compromise bacterial membranes and inhibit enzymatic functions. *K. longiflora*, on the other hand, contains alkaloids or other bioactive molecules that act on distinct bacterial pathways. Their combined effects may enhance antibacterial activity. This aligns with the findings of [45], who demonstrated that phenolics and alkaloids can interact synergistically to improve bacterial inhibition. Nevertheless, the proposed phytochemical interactions remain tentative in the present study because the individual compounds and their concentrations were not characterised or quantified.

The results for *S. mutans* (Table 6) exhibited both indifferent and antagonistic interactions. Notably, combinations involving *E. undulata* with *K. longiflora*, *D. elata*, and *D. senecioides* showed antagonistic effects with high FIC values. This suggests that the combined extracts may interfere with each other’s antibacterial mechanisms, potentially leading to reduced bioavailability or counteracting effects of active compounds. Aloe comprises anthraquinones, flavonoids, and polysaccharides, whereas *K. longiflora* is believed to contain alkaloids and other bioactive compounds. Certain interactions between these constituents may counteract each other’s antibacterial effects, leading to reduced overall activity. Research by [46] found that polyphenols and alkaloids can occasionally display antagonistic interactions, likely due to competition for binding sites on bacterial enzymes.

All plant combinations exhibited antagonistic effects against *P. gingivalis*, indicating that the combined extracts diminished antibacterial activity rather than enhancing it (Table 7). This antagonism may stem from intricate phytochemical interactions, such as tannins binding with alkaloids or flavonoids, which could reduce their effectiveness. Furthermore, *P. gingivalis* is a highly resilient anaerobic pathogen, likely requiring more potent or specifically targeted antibacterial agents. The authors of [47] found that elevated tannin concentrations could hinder the antibacterial properties of flavonoids in oral pathogens, potentially explaining the observed reduction in efficacy. In contrast to the predominantly antagonistic interactions observed against *P. gingivalis*, selected combinations showed enhanced activity against *P. aeruginosa*. Specifically, the combination of *E. undulata* and *K. longiflora* exhibited a synergistic effect (FICI = 0.15), while *E. undulata* + *D. elata* showed an additive effect (FICI = 0.62), and *E. undulata* + *S. senecioides* exhibited a synergistic effect (FICI = 0.08) (Table 8). These findings are consistent with previous reports demonstrating that combinations of plant extracts can produce synergistic effects against various pathogens, including *P. aeruginosa* [45]. However, most combinations against *E. faecalis* were neither synergistic nor additive, although selected combinations showed additive or synergistic interactions (Table 9). This variation suggests that the interaction between plant extracts is dependent on the specific bacterial strain and the chemical composition of the extracts rather than simply on combining two extracts. Similarly, ref. [47] reported that extracts containing shared phytochemicals, such as those found in *S. didymobotrya* and *C. sinensis*, could demonstrate synergistic effects, suggesting that the concentration and composition of bioactive constituents may influence antibacterial interactions. However, because the present study did not characterise or quantify individual phytochemical constituents, the proposed relationship between specific compounds and the observed synergistic or additive effects remains speculative. Therefore, the synergistic effects observed against *P. aeruginosa* should be interpreted as experimental interaction effects rather than definitive evidence of phytochemical synergy.

Importantly, the combinatorial findings revealed specific synergistic interactions that were distinct from additive, indifferent and antagonistic outcomes. Against *P. aeruginosa*, synergistic activity was observed for *E. undulata* combined with *K. longiflora* (FICI = 0.15) and *S. senecioides* (FICI = 0.08), with the latter representing the strongest synergistic interaction identified in the present study. In addition, *A. greatheadii* combined with *E. undulata* exhibited a synergistic effect against *E. faecalis* (FICI = 0.37). These findings suggest that selected combinations may enhance antibacterial efficacy through complementary interactions between bioactive constituents. However, synergy was not observed for all combinations. For example, *E. undulata* combined with *D. elata* demonstrated an additive effect against *P. aeruginosa* (FICI = 0.62), while several other combinations were classified as indifferent or antagonistic. This indicates that the antibacterial effects of plant combinations are dependent on the specific extract combination and bacterial species, rather than being universally enhanced by combining medicinal plants.

Inhibition of the initial cell attachment stage is crucial for preventing biofilm development. In this study, >50% inhibition was considered good activity, <50% indicated limited activity, while negative values indicated a potential pro-biofilm effect. The percentage inhibition results demonstrated that antibiofilm activity varied according to the plant extract, bacterial strain, concentration and stage of biofilm development.

During initial cell attachment, *E. undulata* methanol extract demonstrated over 60% inhibition of *P. gingivalis*, even at sub-MIC concentrations, suggesting it interferes with critical processes like cell attachment (Figure 4). Studies by [45] also found similar plant and marine extracts inhibited bacterial attachment and biofilm formation at sub-MIC concentrations. However, the *A. greatheadii* extract also exhibited over 50% inhibition against *S. mutans* attachment at all concentrations (0.32, 0.62 and 2.52 µg/mL), including sub-MIC, making it effective in preventing caries development by disrupting the attachment process. A study by [48,49] also supported this, highlighting its potential in oral care products. *K. longiflora* also exhibited good activity against *S. aureus*, particularly at higher concentrations. These findings suggest that some extracts may interfere with early bacterial adhesion, thereby limiting the establishment of biofilms. These extracts may disrupt bacterial surface proteins or biofilm matrix formation. Similar inhibition of bacterial attachment by plant-derived extracts at sub-MIC concentrations has been reported [50]. The IC_50_ results further supported differences in antibiofilm potency during this stage. *D. elata* showed the lowest IC_50_ against *S. mutans* (0.001 mg/mL), while *S. senecioides* showed a low IC_50_ against *S. aureus* (0.002 µg/mL), indicating that relatively low concentrations were sufficient to achieve 50% inhibition. Thus, although percentage inhibition reflects the extent of inhibition at the tested concentrations, the IC_50_ provides an indication of the concentration-dependent potency of the extracts.

During biofilm prevention, *A. greatheadii* exhibited strong inhibition against *P. gingivalis* biofilm formation of approximately 90–95% (Figure 5)*. Euclea undulata* (0.075–0.6 µg/mL), *A. greatheadii* (0.320 µg/mL–2.52 µg/mL), and *S. senecioides* (0.075–0.62 µg/mL) effectively inhibited *Streptococcus mutans* across all tested concentrations at the biofilm prevention stage for these plants. These extracts not only inhibited bacterial growth but also prevented early biofilm development, making them potential candidates for oral care products. The corresponding IC_50_ values indicated strong potency of *E. undulata* against *S. mutans* (0.004 µg/mL) and *D. elata* against *P. aeruginosa* (0.05 µg/mL). The low IC_50_ values suggest that these extracts were capable of producing 50% inhibition at relatively low concentrations, complementing the high percentage inhibition observed in the prevention assay. Findings align with studies by [51], which reported similar antibiofilm activity of *S. senecioides* and *Aloe vera* against *S. mutans*. The antibiofilm findings of the present study are also broadly consistent with previous reports demonstrating that medicinal plant extracts can interfere with bacterial attachment and biofilm formation. The observed activity varied according to the bacterial species and stage of biofilm development, as also reflected by the different MBIC_50_/IC_50_ values obtained in the present study (Table 10). This variability may be related to differences in bacterial susceptibility and the ability of extract constituents to interfere with adhesion, extracellular polymeric substance formation and biofilm maturation [50,51].

In contrast, *S. aureus* showed limited inhibition by the extracts, with some treatments producing negative values at sub-MIC concentrations. This may indicate that low concentrations can produce stress responses that favour biofilm development. Similar findings were reported by [50], reinforcing the idea that plant-derived compounds at low doses might unintentionally stimulate *S. aureus* biofilm formation instead of suppressing it.

For established biofilm eradication, *A. greatheadii* exhibited strong inhibition over 50% of mature biofilms at all tested concentrations against *S. mutans* (0.30–2.52 µg/mL) and *P. aeruginosa* (0.012–1.240 µg/mL), while *S. senecioides* showed notable activity against *P. gingivalis* (0.30 and 0.60 µg/mL) and *E. faecalis* (0.63,1.26 and 2.52 µg/mL) (Figure 6). This suggests the presence of bioactive compounds that disrupt the extracellular polymeric substance (EPS) matrix and enhance bacterial susceptibility. *Klenia longiflora* also showed good inhibition of established *S. aureus* biofilm at higher concentrations. The IC_50_ results further demonstrated the potency of these extracts, with *A. greatheadii* showing particularly low IC_50_ values against *S. mutans* (7.91 × 10^−6^ µg/mL) and *P. aeruginosa* (0.006 µg/mL), while *S. senecioides* showed an IC_50_ of 0.06 µg/mL against *E. faecalis*. These results indicate that the extracts showing high percentage inhibition against established biofilms also exhibited strong potency at relatively low concentrations. This is likely due to bioactive compounds that interfere with microbial adhesion and biofilm structure [52]; *Quercus robur* exhibited strong antibacterial activity (MIC < 1 µg/mL), while *Coffea arabica* reduced *S. aureus* biofilm growth by 91.4%, highlighting their potential for bacterial inhibition and biofilm control. In contrast, other extracts failed to eradicate mature biofilms, possibly due to their inability to penetrate the biofilm matrix or disrupt bacterial survival mechanisms.

Taken together, the present findings support previous evidence that medicinal plant extracts can exhibit both antibacterial and antibiofilm properties, although the magnitude of activity is dependent on the extract, bacterial strain and biofilm stage (Table 11). The antibacterial and antibiofilm activities observed are consistent with previous reports, although activity varied with plant species, extraction solvent and bacterial strain. *Euclea undulata* exhibited the strongest antibacterial activity, with MIC values of 0.15 to >2.5 µg/mL, including notable activity against *S. mutans* and *P. gingivalis* (MIC = 0.15 µg/mL). This supports previous reports linking the antimicrobial activity of *E. undulata* and related *Euclea* species to naphthoquinones such as 7-methyljuglone, diospyrin and isodiospyrin [15,53]. *Aloe greatheadii* also exhibited antibacterial and antibiofilm activity across prevention, initial attachment and eradication stages. Previous studies reported MIC values of 0.067–0.54 mg/mL against *Enterococcus faecalis* [16,48,49]. Differences from the present findings may reflect variations in geographical origin, extraction methods, phytochemical composition and bacterial susceptibility. The antibiofilm findings indicate that antibacterial potency was not always proportional to biofilm inhibition. *E. undulata* showed >60% inhibition of *P. gingivalis* initial attachment at sub-MIC concentrations, suggesting interference with adhesion or early biofilm formation. *A. greatheadii* also demonstrated consistent antibiofilm activity despite variations in MIC values, highlighting the distinct mechanisms underlying biofilm formation and planktonic growth. Previous phytochemical studies have identified phenolic acids, flavonoids, alkaloids, tannins, terpenoids, steroids and other secondary metabolites in the investigated plants. *Kleinia longiflora* contains lupeol, isorhamnetin glycosides and stigmasterol, while *S. senecioides* contains phenolics, flavonoids, alkaloids, glycosides, terpenoids and steroids [17,49,54,55]. *Drimia elata* has also been associated with bufadienolides, flavonoids, tannins, saponins, steroids and terpenoids and reported antibacterial activity against several pathogens [54,56]. These metabolites may contribute to the observed activities; however, as compound identification and quantification were not performed, direct associations with individual compounds cannot be established. The combinatorial findings revealed specific synergistic interactions against *P. aeruginosa*, involving *E. undulata* with *K. longiflora* (FICI = 0.15) and *S. senecioides* (FICI = 0.08), as well as *A. greatheadii* with *E. undulata* against *E. faecalis* (FICI = 0.37). In contrast, other combinations exhibited additive, indifferent or antagonistic effects, indicating that the efficacy of plant combinations is dependent on the specific extracts and bacterial species [55]. While previous studies have mainly focused on the individual antibacterial properties and phytochemical composition of these plants, limited information is available regarding their combinatorial interactions. The present study demonstrated specific synergistic effects between *E. undulata* and *K. longiflora* (FICI = 0.15) and *S. senecioides* (FICI = 0.08) against *P. aeruginosa*, as well as between *A. greatheadii* and *E. undulata* against *E. faecalis* (FICI = 0.37). These findings extend previous knowledge by demonstrating the potential of selected medicinal plant combinations to enhance antibacterial activity. However, synergy was not observed in all combinations, with *E. undulata* and *D. elata* exhibiting an additive effect against *P. aeruginosa* (FICI = 0.62), while other combinations were indifferent or antagonistic. Further LC-MS-based phytochemical profiling and mechanistic studies would therefore be required to identify the compounds responsible for these activities and to establish the mechanisms underlying the observed extract interactions. The potential application of the medicinal plant extracts in oral healthcare products requires consideration of both antimicrobial efficacy and safety. In a related study, ref. [57] evaluated the cytotoxicity and selectivity of the same plants and corresponding extracts used in the present investigation, providing complementary safety information for their proposed dental applications. These findings support further evaluation of the extracts as potential ingredients in oral healthcare formulations, particularly at concentrations demonstrating effective antibacterial and antibiofilm activity.

Overall, the percentage inhibition and IC_50_ results provide complementary measures of antibiofilm activity. Percentage inhibition demonstrates the extent of biofilm inhibition at the concentrations tested, whereas IC_50_ indicates the concentration required to achieve 50% inhibition; therefore, lower IC_50_ values indicate greater potency. Taken together, the results demonstrate strain- and biofilm-stage-dependent activity, with *A. greatheadii* showing particularly strong activity against established biofilms, while other extracts demonstrated greater potency against specific organisms or earlier stages of biofilm development.

**Table 11:** Comparison of antibacterial, combinatorial and antibiofilm activities of selected medicinal plants with previous studies.

Plant Extract	Test Bacteria	MIC in Present Study (µg/mL)	FIC/FICI in Present Study	Antibiofilm Activity in the Present Study	Bioactive Compounds Reported Previously	Comparison with Previous Studies	Reference
*A. greatheadii*	*P. aeruginosa*, *E. faecalis*, *S. aureus*, *S. mutans*, *P. gingivalis*	0.31–>2.5	Synergistic with *E. undulata* against *E. faecalis* (FICI = 0.37); additive with selected combinations	Activity observed during biofilm prevention, initial attachment and established biofilm eradication	Phenolic acids, phenolics, phytosterols, fatty acids and indoles	Reported antibacterial activity of *A. greatheadii* against *E. faecalis*, with MIC values of 0.067–0.54 mg/mL depending on geographical origin. Identified phenolic acids, including 4-hydroxyphenylacetic acid, protocatechuic acid, p-coumaric acid and caffeic acid, together with campesterol and β-sitosterol.	[17,18,55]
*E. undulata*	Same five bacteria	0.15–>2.5	Synergistic with *K. longiflora* (FICI = 0.15) and *S. senecioides* (FICI = 0.08) against *P. aeruginosa;* additive with selected combinations against *E. faecalis*	Demonstrated activity during biofilm prevention, initial attachment and established biofilm eradication	7-methyljuglone, diospyrin, isodiospyrin and other naphthoquinones	Previous phytochemical studies reported 7-methyljuglone and diospyrin from the roots of *E. undulata*, with isodiospyrin also reported from the fruits. Antimicrobial activity has been associated with naphthoquinones reported in *Euclea species*.	[53,58]
*K. longiflora*	Same five bacteria	0.63–>2.5	Synergistic with *E. undulata* against *P. aeruginosa* (FICI = 0.15); additive with selected combinations against *E. faecalis*	Antibiofilm activity observed at different stages	Lupeol, isorhamnetin glycoside, stigmasterol, β-eudesmol, ambrosin and other terpenoids	Reported terpenoids, tannins, alkaloids and flavonoids in *K. longiflora*, and isolated lupeol, an isorhamnetin glycoside and stigmasterol. GC-MS analysis also identified β-eudesmol, lupeol, ambrosin and α-panasinsen among the major constituents.	[16,48]
*S. senecioides*	Same five bacteria	0.15–>2.5	Synergistic with *E. undulata* against *P. aeruginosa* (FICI = 0.08)	Antibiofilm activity observed	Phenolics, flavonoids, alkaloids, glycosides, terpenoids and steroids; flavonoid, steroidal and triterpenoid glycosides	Reported phenolics, flavonoids, alkaloids, glycosides, terpenoids and steroids in *Dicerocaryum senecioides*, with flavonoid, steroidal and triterpenoid glycosides identified by TLC.	[59,60]
*D. elata*	Same five bacteria	0.63–>2.5	Additive with *E. undulata* against *P. aeruginosa* (FICI = 0.62); other combinations mainly indifferent/antagonistic	Antibiofilm activity observed	Bufadienolides, aromatic acids, flavonoids, tannins, saponins, steroids and terpenoids	Reported antibacterial activity of *D. elata* extracts against *S. aureus*, *E. faecalis*, *E. coli* and *P. aeruginosa*, with MIC values ranging from 0.2 to 12.5 µg/mL. Other studies summarised reported MIC values from 0.8 to >12.5 µg/mL, exhibiting variation according to plant part, extraction solvent and bacterial strain.	[54,56]

## 5. Conclusions

The current study demonstrated that HPTLC fingerprinting revealed clear differences in the phytochemical profiles of the five medicinal plants, which complemented the quantitative phytochemical analysis. *E. undulata* showed high levels of phenolics, flavonoids and tannins, corresponding with its notable antibacterial activity, including an MIC of 0.15 µg/mL against both *S. mutans* and *P. gingivalis*. The antibacterial activity varied according to the plant extract and bacterial strain. *A. greatheadii* demonstrated particularly promising antibiofilm activity across initial cell attachment, biofilm prevention and established biofilm eradication. The combination studies showed variable interactions, with most combinations being indifferent or antagonistic, while selected combinations exhibited additive or synergistic effects. Notably, *E. undulata* + *K. longiflora* exhibited a synergistic effect against *P. aeruginosa*. Overall, the findings support the potential of these medicinal plants as sources of natural antibacterial and antibiofilm agents. However, although phytochemical differences were demonstrated, the individual compounds responsible for the observed activities were not identified or quantified in the present study. Further phytochemical profiling, compound isolation and mechanistic studies are therefore required to establish the constituents responsible for these activities and their potential application in oral healthcare. Future studies should make comparisons with standard phenolics, tannins and flavonoids against test bacteria, and then the results should be compared with gentamicin.

## Figures and Tables

**Table 1:** Medicinal plants used by Bapedi traditional healers to treat and prevent oral infections and related symptoms in the Limpopo Province, South Africa.

Scientific Name	Local Name	Family Name	Voucher Specimen	Oral Health Application
*Kleinia longiflora* DC.	Mbalati (Tsonga) Sebalo (Sepedi) Candle plant (English)	*Asteraceae*	UNIN1220298	Traditionally used for mouth sores and general inflammation [16]
*Aloe greatheadii* var. *davyana* Schönland.	Tshikhopha (Tshivenda) Spotted Aloe (English)	Asphodelaceae	UNIN1220301	Gel used to soothe oral ulcers and gum inflammation [17,18]
*Drimia elata* Jacq. Ex Willd.	Sekanama (Sepedi) Satin Squill (English)	Hyacinthaceae	UNIN1220300	Bulbs used for inflammation and occasionally in tooth remedies [21,22]
*Euclea undulata* Thunb.	Common Guarri (English), Mopipi (Setswana)	Ebenaceae	UNIN1220297	Used to strengthen teeth and treat toothache and oral sores [15,16]
*Sesamum senecioides* (Klotzsch) Byng and	Devil’s thorn, boot protectors, stud plant (English) Museto (Tshivenda)	Pedaliaceae	UNIN1220296	Used for treating toothache and gum infections [17,21,22]

**Table 3:** Quantification of five plant extracts’ total phenolic, flavonoids, and tannin content.

Plant Name	Solvents	Total Phenolic Content (mg of GAE/g)	Total Flavonoid Content (mg of QE/g)	Total Tannin Content (mg of GAE/g)
*Klenia longiflora*	PE	191.50 ± 5.39 ^c,d,e,f^	21.98 ± 4.42 ^b,c,d^	736.75 ± 5.25 ^i^
	DCM	194.96 ± 3.03d ^e,f,g^	7.06 ± 5.65 ^a,b,c^	531.08 ± 4. 97 ^g^
	METH	216.74 ± 7.19 ^f,g,h^	10.51 ± 3.91 ^a,b,c^	940.75 ± 4.71 ^j^
	Water	163. 69 ± 8.93 ^a,b^	0.98 ± 0.57 ^a,b^	257.75 ± 3.86 ^d^
*Drimia elata*	PE	172.32 ± 477 ^a,b,c,d^	2.38 ± 1,87 ^a,b^	200.75 ± 8.35 ^c^
	DCM	213.61 ± 9.10 ^e,f,g,h^	5.55 ± 4.64 ^a,b,c^	318.08 ± 10.87 ^e^
	METH	229.95 ± 3.21 ^h,i^	1.14 ± 0.7 ^a,b^	214.75 ± 1.66 ^c^
	Water	160.99 ± 4.06 ^a,b^	0.42 ± 0.00 ^a^	81.75 ± 1.26 ^a^
*Sesamum senecioides*	PE	202.34 ± 0.10 ^e,f,g,h^	0.89 ± 0 ^a,b^	1237.42 ± 8.31 ^l^
	DCM	225.18 ± 5.52 ^h^	15.58 ± 1.84 ^a,b,c,d^	425.42 ± 7.5 ^f^
	METH	185. 52 ± 0.41 ^b,c,e^	19.92 ± 2.68 ^a,b,c,d^	2324.42 ± 9.41 ^m^
	Water	169.67 ± 8.89 ^a,b,c^	0.49 ± 0.38 ^a^	140.75 ± 2.64 ^b^
*Aloe greatheadii*	PE	196.31 ± 7.68 ^d,e,f,g^	12.63 ± 0.20 ^a,b,c,d^	220.75 ± 2 ^c^
	DCM	222.09 ± 4.70 ^g,h^	17.92 ± 2.31 ^a,b,c,d^	539.08 ± 7.0 ^g^
	METH	253.89 ± 1.74 ^i,j^	7.07 ± 6.70 ^a,b,c^	673.42 ± 5.5 ^h^
	Water	153.53 ± 6.30 ^a^	0.28 ± 0.23 ^a^	209.75 ± 1 ^c^
*Euclea undulata*	PE	210.48 ± 1.23 ^e,f,g,h^	31.26 ± 2.76 ^d^	2400.74 ± 8.31 ^n^
	DCM	226.77 ± 7.62 ^h^	25.98 ± 2.54 ^c,d^	1146.75 ± 12.33 ^l^
	METH	269.84 ± 2.76 ^j^	2.39 ± 2.35 ^a,b^	1251.75 ± 7.28 ^l^
	Water	377.63 ± 2.25 ^k^	5.81 ± 0.94 ^a,b,c^	1149.42 ± 2.15 ^k^

Keys: PE: Petroleum ether, DCM: dichloromethane, METH: methanol. In each column, average values with the same letters have a statistically insignificant difference (Tukey’s multiple comparison test), *p* < 0.05.

**Table 5:** Combinational effects of various plants on antibacterial activity against *Staphylococcus aureus*.

Combinations	MIC (µg/mL)	FIC Values		FIC Index	Outcomes
		A	B		
*A. greatheadii* + *K. longiflora*	1.25	0.5	1.98	2.48	Indifferent
*A. greatheadii* + *D. elata*	2.5	1	1	2	Indifferent
*A. greatheadii* + *S. senecioides*	0.31	0.12	1	1.124	Indifferent
*A. greatheadii* + *E. undulata*	0.31	0.12	0.49	0.62	Additive
*E. undulata* + *K. longiflora*	0.31	0.49	0.49	0.98	Additive
*E. undulata* + *D. elata*	1.25	1.98	0.5	2.48	Indifferent
*E. undulata* + *S. senecioides*	0.63	1	2.03	3.03	Indifferent
*D. elata* + *K. longiflora*	2.5	1	3.97	4.97	Antagonistic
*D. elata* + *S. senecioides*	1.25	0.5	4.03	4.53	Antagonistic
*K. longiflora* + *S. senecioides*	0.63	1	2.03	3.03	Indifferent

Key: FIC—fractional inhibitory concentration.

**Table 6:** Combinational effects of various plant extracts on antibacterial activity against *Streptococcus mutans*.

Combinations	MIC (µg/mL)	FIC Values		FIC Index	Outcomes
		A	B		
*A. greatheadii* + *K. longiflora*	1.25	1.98	1.98	3.97	Indifferent
*A. greatheadii* + *D. elata*	1.25	1.98	1.98	3.97	Indifferent
*A. greatheadii* + *S. senecioides*	0.31	0.49	1	1.49	Indifferent
*A. greatheadii* + *E. undulata*	0.15	0.24	1	1,24	Indifferent
*E. undulata* + *K. longiflora*	1.25	8.33	1.98	10.32	Antagonistic
*E. undulata* + *D. elata*	1.25	8.33	1.98	10.32	Antagonistic
*E. undulata* + *S. senecioides*	0.63	4.2	2.03	6.23	Antagonistic
*D. elata* + *K. longiflora*	1.25	1.98	1.98	3.97	Indifferent
*D. elata* + *S. senecioides*	1.25	1.98	4.03	6.02	Antagonistic
*K. longiflora* + *S. senecioides*	1.25	1.98	4.032	6.02	Antagonistic

Key: FIC—fractional inhibitory concentration.

**Table 7:** Combinational effects of various plant extracts on antibacterial activity against *Porphyromonas gingivalis*.

Combinations	MIC (µg/mL)	FIC Values		FIC Index	Outcomes
		A	B		
*A. greatheadii* + *K. longiflora*	2.5	8.07	2	10.07	Antagonistic
*A. greatheadii* + *D. elata*	2.5	8.07	3.97	12.03	Antagonistic
*A. greatheadii*+ *S. senecioides*	0.63	2.03	4.2	6.23	Antagonistic
*A. greatheadii* + *E. undulata*	0.63	2.03	4.2	6.23	Antagonistic
*E. undulata* + *K. longiflora*	0.63	4.2	0.5	4.70	Antagonistic
*E. undulata* + *D. elata*	1.25	8.33	1.98	10.32	Antagonistic
*E. undulata* + *S. senecioides*	0.63	4.2	4.2	8.4	Antagonistic
*D. elata* + *K. longiflora*	2.5	3.97	2	5.97	Antagonistic
*D. elata* + *S. senecioides*	0.63	1	4.2	5.2	Antagonistic
*K. longiflora* + *S. senecioides*	1.25	1	8.3	9.3	Antagonistic

Key: FIC—fractional inhibitory concentration.

**Table 8:** Combinational effects of various plant extracts on antibacterial activity against *Pseudomonas aeruginosa*.

Combinations	MIC (µg/mL)	FIC Values		FIC Index	Outcomes
		A	B		
*A. greatheadii* + *K. longiflora*	1.25	4.03	0.5	4.53	Antagonistic
*A. greatheadii* + *D. elata*	1.25	4.03	0.5	4.53	Antagonistic
*A. greatheadii*+ *S. senecioides*	0.31	1	0.49	1.49	Indifferent
*A. greatheadii* + *E. undulata*	0.31	1	0.49	1.49	Indifferent
*E. undulata* + *K. longiflora*	0.075	0.12	0.03	0.15	Synergistic
*E. undulata* + *D*. *elata*	0.31	0.49	0.12	0.62	Additive
*E. undulata* + *S. senecioides*	0.039	0.06	0.02	0.08	Synergist
*D. elata* + *K. longiflora*	2.5	1	1	2	Indifferent
*D. elata* + *S. senecioides*	1.25	0.5	1.98	2.48	Indifferent
*K. longiflora* + *S. senecioides*	0.63	0.25	1	1.25	Indifferent

Key: FIC—fractional inhibitory concentration.

**Table 9:** Combinational effects of various plant extracts on antibacterial activity against *Enterococcus faecalis*.

Combinations	MIC (µg/mL)	FIC Values		FIC Index	Outcomes
		A	B		
*A. greatheadii*+ *K. longiflora*	1.25	0.5	0.5	1	Additive
*A. greatheadii* + *D. elata*	0.63	0.25	0.25	0.50	Additive
*A. greatheadii* + *S. senecioides*	0.31	0.12	0.49	0.62	Additive
*A. greatheadii* + *E. undulata*	0.31	0.12	0.25	0.37	Synergistic
*E. undulata* + *K. longiflora*	0.63	0.50	0.25	0.76	Additive
*E. undulata* + *D. elata*	0.63	0.50	0.25	0.76	Additive
*E. undulata* + *S. senecioides*	0.63	0.50	1	1.50	Indifferent
*D. elata* + *K. longiflora*	2.5	1	1	2	Indifferent
*D. elata* + *S. senecioides*	0.63	0.25	1	1.25	Indifferent
*K. longiflora* + *S. senecioides*	1.25	0.5	1.98	2.48	Indifferent

Key: FIC—fractional inhibitory concentration.

**Table 10:** Minimum Biofilm Inhibitory Concentration (MBIC50) of the methanol extract.

MBIC_50_ (µg/mL) against biofilm prevention					
Extracts	*S. mutans*	*S. aureus*	*P. gingivalis*	*P. aeruginosa*	*E. faecalis*
*E. undulata*	0.004 ± 4.98	0.89 ± 20.01	0.98 ± 26.38	1.26 ± 30.57	1.52 ± 37.52
*K. longiflora*	0.88 ± 19.64	0.93 ± 19.33		-	-
*A. greathedii*	0.89 ± 7.93	-	1.13 ± 29.66	27.33 ± 29.35	-
*S. senecoides*	1.37 ± 5.97	9.39 ± 16.71	1.59 ± 16.08	0.05± 17.83	1.96 ± 9.33
*D. elata*	0.31 ± 9.6	-	5.31 ± 0.01	-	-
Gentamicin	1.08 ± 0.29	0.03 ± 3.36	0.01 ± 0.86	0.34 ± 14.90	0.63 ± 2.01
MBIC_50_ (µg/mL) against Initial Cell Attachment					
Extracts	*S. mutans*	*S. aureus*	*P. gingivalis*	*P. aeruginosa*	*E. faecalis*
*E. undulata*	1161 ± 1.95	5.42 ± 4.56	0.04 ± 9.68	3.93 ± 4.14	-
*K. longiflora*	11.17 ± 21.24	1.41 ± 7.79	-	-	-
*A. greatheadii*	4.46 ± 3.25	-	206 ± 22.19	9.92 ± 0.01	-
*S. senecoides*	0.01 ± 11.02	0.002 ± 7.81	7117 ± 13.98	1.03 ± 6.32	1.44 ± 5.33
*D. elata*	0.001 ± 6.14	-	1.14 ± 19.29	-	-
Gentamicin	0.02 ± 2.15	0.65 ± 3.43	1.83 ± 1.26	0.03 ± 4.02	0.002 ± 2.32
MBIC_50_ (µg/mL) against Eradication of biofilm					
Extracts	*S. mutans*	*S. aureus*	*P. gingivalis*	*P. aeruginosa*	*E. faecalis*
*E. undulata*	0.54 ± 2.035	1.99 ± 6.40	1.00 ± 2.01	1.96 ± 7.57	-
*K. longiflora*	3.91 ± 7.342	0.99 ± 5.62	-	-	-
*A. greathedii*	0.06 ± 1.28	-	0.87 ± 5.96	0.006 ± 5.25	-
*S. senecoides*	1.44 ± 5.57	71 ± 16.36	1.34 ± 13.86	2.14 ± 11.83	0.06± 14.29
*D. elata*	10.69 ± 1.45	-	1.53 ± 6.57	-	-
Gentamicin	0.001 ± 0.01	1.23 ± 2.69	0.04 ± 3.44	0.002 ± 11.02	1.12 ± 4.78

Key: - not detected.

## Data Availability

The original contributions presented in this study are included in the article. Further inquiries can be directed to the corresponding author.

## Abbreviations

The following abbreviations are used in this manuscript:

AlCl_3_	aluminium chloride
ANOVA	analysis of variance
ATCC	American Type Culture Collection
BEA	benzene/ethanol/ammonium hydroxide
CEF	chloroform/ethyl acetate/formic acid
CFU/mL	colony-forming units per millilitre
CV	crystal violet
DCM	dichloromethane
EMW	ethyl acetate/methanol/water
EPS	extracellular polymeric substance
FIC	fractional inhibitory concentration
GAE/g	gallic acid equivalent per gram
HPTLC	high-performance thin-layer chromatography
INT	p-iodonitrotetrazolium chloride
MIC	minimum inhibitory concentration
NaNO_2_	sodium nitrite
NaOH	sodium hydroxide
OD	optical density
PE	petroleum ether
QE/g	quercetin equivalents per gram
Rf	retention factor
SDHC,	Sundy hammer crusher
STIs	sexually transmitted infections
TPC	total phenolic content
UV/VIS	ultraviolet-visible.
